# Imidazoquinolines with improved pharmacokinetic properties induce a high IFNα to TNFα ratio *in vitro* and *in vivo*

**DOI:** 10.3389/fimmu.2023.1168252

**Published:** 2023-06-20

**Authors:** Manuel Keppler, Simon Straß, Sophia Geiger, Tina Fischer, Nadja Späth, Thilo Weinstein, Anna Schwamborn, Jamil Guezguez, Jan-Hinrich Guse, Stefan Laufer, Michael Burnet

**Affiliations:** ^1^ Synovo GmbH, Tübingen, Germany; ^2^ Pharmaceutical Chemistry, Institute for Pharmaceutical Sciences, Eberhard Karls University Tübingen, Tübingen, Germany

**Keywords:** TLR7, TLR8, resiquimod, imidazoquinoline, Interferon a (IFNa), macrolide, cytokine spectrum, pharmacokinetics

## Abstract

TLR Agonists have promising activity in preclinical models of viral infection and cancer. However, clinical use is only in topical application. Systemic uses of TLR-ligands such as Resiquimod, have failed due to adverse effects that limited dose and thus, efficacy. This issue could be related to pharmacokinetic properties that include fast elimination leading to low AUC with simultaneously high c_max_ at relevant doses. The high c_max_ is associated with a sharp, poorly tolerated cytokine pulse, suggesting that a compound with a higher AUC/c_max_-ratio could provide a more sustained and tolerable immune activation. Our approach was to design TLR7/8-agonist Imidazoquinolines intended to partition to endosomes *via* acid trapping using a macrolide-carrier. This can potentially extend pharmacokinetics and simultaneously direct the compounds to the target compartment. The compounds have hTLR7/8-agonist activity (EC50 of the most active compound in cellular assays: 75-120 nM hTLR7, 2.8-3.1 µM hTLR8) and maximal hTLR7 activation between 40 and 80% of Resiquimod. The lead candidates induce secretion of IFNα from human Leukocytes in the same range as Resiquimod but induce at least 10-fold less TNFα in this system, consistent with a higher specificity for human TLR7. This pattern was reproduced *in vivo* in a murine system, where small molecules are thought not to activate TLR8. We found that Imidazoquinolines conjugated to a macrolide or, substances carrying an unlinked terminal secondary amine, had longer exposure compared with Resiquimod. The kinetics of pro-inflammatory cytokine release for these substances *in vivo* were slower and more extended (for comparable AUCs, approximately half-maximal plasma concentrations). Maximal IFNα plasma levels were reached 4 h post application. Resiquimod-treated groups had by then returned to baseline from a peak at 1 h. We propose that the characteristic cytokine profile is likely a consequence of altered pharmacokinetics and, potentially, enhanced endosomal tropism of the novel substances. In particular, our substances are designed to partition to cellular compartments where the target receptor and a distinct combination of signaling molecules relevant to IFNα-release are located. These properties could address the tolerability issues of TLR7/8 ligands and provide insight into approaches to fine-tune the outcomes of TLR7/8 activation by small molecules.

## 1 Introduction

The term immunotherapy is mostly associated with the treatment of cancer by checkpoint inhibitors, cell-based approaches or vaccinations (1). In a broader sense, immunotherapy describes therapeutic concepts aimed at modulating the host immune system to treat conditions related to neoplasia and infection as well as autoimmune conditions (2–4). The possibility of harnessing the diverse defense mechanisms of the immune system itself circumvents some of the limitations of pathogen- or neoplasm-directed pharmaceuticals, particularly the development of resistance to those drugs by mutations in their molecular targets. Correspondingly, immune activating therapies could be useful for infectious diseases for which a drug specifically targeting the pathogen itself is not yet available (4–6). One of the first treatments relying on immune activation to interfere with an ongoing infection or neoplasm was the use of recombinant Interferon alpha (IFNα) in the treatment of Hairy Cell Leukemia, Hepatitis B and Hepatitis C (before HBV/HCV were identified) (7–9). Indeed, recombinant IFNα was the first approved immunotherapeutic agent and the most thoroughly clinically characterized immune stimulant. It has been used for decades as a treatment of various viral diseases and cancers (8). While there has been a considerable focus on checkpoint blockade *via* antibodies targeting the PD1/PD-L1-axis (10), stimulation of the immune system through activation of pattern recognition receptors (PRRs) and particularly Toll-like-receptors (TLRs) is another promising concept that has been widely investigated, especially in dermatological cancers (11–13). In contrast to immunotherapies focused on blocking of inhibitory receptors and thus overcoming immunosuppression, agonists to PRRs activate immune response through increasing the expression of surface-bound and secreted mediators of inflammation making this stimulatory approach more similar to the direct use of recombinant cytokines such as IFNα as therapeutic agents (13–17).

Toll-like receptors are membrane-integral PRRs with varied representation in different species – 10 subtypes have been identified in humans and 12 in mice. Regardless of species, TLRs can generally be subdivided based on the orientation of their ectodomains either towards the extracellular space or the luminal space of endosomal vesicles. In humans, TLR1/2/4/5/6/10 are localized in the plasma membrane while TLR3/7/8/9 are restricted to vesicular membranes. TLR10 is not present in mice but TLR11/12/13 are and all localize to membranes of intracellular compartments (18). Ligand binding results in the formation of receptor dimers and recruitment of adapter proteins containing a TIR domain which vary with receptor type. The signaling cascade of all TLRs except for TLR3 uses the common adapter MyD88 to subsequently activate NFκB and IRF1/3/5/7/8 depending on receptor and cell type, with activation of NFκB and IRF5 being linked to the induction of pro-inflammatory cytokines and IRF1/3/5/7/8 having a role in regulating expression of type I interferons (19–21).

The endosomal nucleic acid-sensing TLRs 7/8/9 are similar in terms of their natural ligands, localization and direct engagement of MyD88. This is in contrast to plasma membrane-localized TLRs which employ additional adapters such as TIRAP and TRAM or TLR3 which uses TRIF as its downstream adapter. TLR7/8/9 can therefore be classified as a sub-family of TLRs (14, 19). TLR7 and 9 are highly expressed in plasmacytoid dendritic cells (pDCs), which play a central role at the interface between the innate and adaptive immune response to viral infections (22). However, their impact on cancer and immune evasion is ambiguous (23, 24). TLR8 is highly expressed in monocytes, macrophages and myeloid dendritic cells (mDCs) (25).

The outcome of intracellular TLR activation can vary considerably as a consequence of receptor expression being limited to specific cell types with characteristic signaling cascades and downstream mediators of inflammation (26). For example, TLR7 mediates release of large amounts of Type I IFN from pDCs. This is in contrast to its role in monocytes, where TLR7 activation by Imiquimod induces secretion of the classical inflammatory cytokines Interleukin-6 (IL6) and -1β (IL1β) but IFNα/β release is instead mediated by TLR8 (22, 27).

Ligand-dependent cytokine expression patterns in a single cell type have been demonstrated for TLR9, for which several classes of synthetic oligodeoxynucleotides (ODNs) with different signaling outcomes have been identified. A distinct induction of either IRF- or NFκB-relayed signaling was originally reported to be dependent on ODN sequence and has later been demonstrated to vary based on the subcellular location of receptor engagement (28–31). In this context TRAF3 and IKKα act as the central mediator of IRF7 phosphorylation and induction of type I IFNs following the activation of TLR9 and 7 (32–35).

Given the similarities in the respective signaling cascades, a similar spatial factor to the outcome of TLR7 activation is plausible, although not yet demonstrated (35). Clear differentiation of the signaling by TLR7 and 8 in native cells will, however, be complicated because there is some overlap also in their ligand preferences. TLR9 signaling, however, can be distinguished because it recognizes unmethylated CpG motifs in DNA (36).

In addition to their ability to bind specific nucleic acid sequences, TLR7/8/9 possess dedicated binding sites for either guanosine (TLR7), uridine (TLR8) or cytosine (TLR9) and simultaneous engagement of both binding sites enhances receptor activation (11). While signaling through TLR9 appears to require a longer oligonucleotide ligand, several synthetic small molecules (mononucleotide analogs) sufficient to activate TLR7/8 are available. The most widely used class of small-molecule TLR7/8-activators are Imidazoquinolines, particularly the most prominent members of this class, Resiquimod (TLR7/8) and Imiquimod (TLR7). Imiquimod is the only FDA-approved agonist to an intracellular TLR to date. Its applications include the topical treatment of basal cell carcinoma or genital warts. Imiquimod probably interacts with other receptors in addition to TLR7 but its efficacy appears to depend on induction of IFNα, tumor necrosis factor α (TNFα), interleukin 12 (IL12) and other pro-inflammatory mediators (15).

The more potent Imidazoquinoline, Resiquimod, originally showed promise in various pre-clinical models of neoplastic or infectious disease (37–39), however, these results have not translated in wider clinical trials. Topical treatment of genital herpes with Resiquimod had encouraging effects in Phase II but not in Phase III (40). In the treatment of chronic Hepatitis C, oral Resiquimod could transiently reduce viral titers but effective doses caused systemic adverse effects consistent with an excess induction of inflammatory cytokines, particularly IFNα (41). Similar adverse events were observed for daily Imiquimod use (42). These adverse effects, often in the form of “flu like” symptoms, limit the systemic use of TLR7 and 8 activators and likewise IFNα. In the latter case, efforts have been made to increase the therapeutic window by PEGylation of recombinant IFNα to improve the pharmacokinetic profile (9), lengthen circulating half-life and allow longer intervals between treatments. Efficacy and adverse event benefits compared with the un-PEGylated cytokine were variable (43, 44).

Following a similar rationale, we hypothesized that the therapeutic index of small molecule TLR agonists like Resiquimod is limited by a range of factors: very steep dose response characteristics (all or nothing), the short half-life and poor tissue distribution necessitating the use of relatively high doses to achieve sufficient activation, the transient stimulation and, correspondingly, the high maximal concentrations relative to the AUC of pro-inflammatory cytokines that are induced.

The transient effects are due to the fact that Resiquimod is unstable and rapidly metabolized. After oral application major metabolites are 6-OH-Resiquimod and 7-OH-Resiquimod *via* CYP1A2; desethyl or N-oxide Resiquimod *via* CYP3A4; or 8-OH-Resiquimod *via* one of both enzymes. Unchanged Resiquimod is only detectable in minor amounts in either urine (< 5%) or feces (< 1%) (45, 46). Imiquimod metabolism is associated with the formation of at least five different monohydroxylated metabolites through CYP1A isoforms (47).

The binding sites of known small-molecule agonists of TLR7 and 8 are each located in the same motif. The aminoquinolyl moiety mediates agonistic receptor binding through stacking effects and through hydrogen bonds (48–51). The butyl side chain interacts hydrophobically with the binding pocket of the receptors. The length of 4 atoms appears optimal for interaction with hTLR7 while heteroatoms in this side group, such as an oxygen in Resiquimod, moderately increase the affinity (49, 50). A smaller contribution to the binding affinity is made by van der Waals interactions of the 2-methylpropan-2-ol side chain (49, 52).

Due to the slightly lower importance of that interaction, the 2-methylpropan-2-ol side chain was chosen as the starting point for modification and linking *via* side groups listed in reaction scheme 1 in **Figure S1** (A1 to A4), which could be further extended by a macrolide (A1-mac and A2-mac; see **Figure S2**). The linking molecules functioned as a spacing between TLR agonistic imidazoquinolinone and macrolide but can also contribute hydrogen bonds as in the 2-methyl-propan-2-ol of Resiquimod. The macrolide site consists of Azithromycin coupled to the TLR binding site at the desosamine *via* an N-methyl iminodiacetyl. Azithromycin provides a high volume of distribution, concentration in immune cells and specifically endo/lysosomes, high exposure to liver, lung and spleen and sub-cellular separation from cytochrome p450 containing organelles (53–56). Compared with other common macrolides, it exhibits increased stability to acids, low hERG affinity, and a greater ability to concentrate in cells due to its dual amines (55, 57–59). Many of these effects are related to its properties as an amphiphilic di-basic compound for which the pk_a_s of the amines correspond well to those required to be neutral during membrane traverse but charged in acidic intracellular compartments. Accumulation of imidazoquinolines and 8-Oxoadenine in endosomal compartments of pDCs has been demonstrated by others and could be a necessary factor in the process of TLR-activation by those compounds (60).

Building on our previous experience with macrolide-derivatives (53, 61), we aimed to prepare TLR7/8 ligands with high exposure to the endo/lysosomal lumen by exploiting the properties of acid trapping in the assumption that amphiphiles would be ideal ligands for endosomal TLRs. Since endosomal tropism has been observed for both macrolides as well as imidazoquinolines, we propose that conjugation of imidazoquinoline TLR7/8 agonists to Azithromycin as a carrier molecule is likely to result in conjugates that accumulate intracellularly as well. We therefore designed imidazoquinoline-ligands in a way that would make them suitable for linkage to carrier molecules, such as Azithromycin, peptides or proteins, i.e. retain activity when conjugated. The ligands described here are intended to partition to their target organelles, either by manipulating the properties of the ligand substituents themselves, or *via* conjugation to Azithromycin, which would dominate the properties of the resulting compound. In parallel we optimized for high *in vivo* stability as well as favorable pharmacokinetic properties following parenteral application. Based on the expected pharmacokinetic properties of our ligands, we expected differences in release kinetics of inflammatory mediators, when compared to other Imidazoquinolines and, correspondingly, changes in maximal and cumulative plasma concentrations of those mediators. What we did not expect were changes in the cytokine spectrum induced by our ligands that may improve tolerability. Here we report the initial characterization of the compounds as small molecules, that may be useful as immune stimulants in cancer and infection.

## 2 Methods

### 2.1 Synthesis and characterization

All chemicals were purchased from commercial sources and used as received. Reaction monitoring was performed *via* mass spectrometry (Finnigan LCQ Deca XP MAX, Software Xcalibur 2.0.7 SP1) and TLC (Merck TLC Silica gel 60 F254). TLC spots were detected with Hanessian’s stain, based on a Cerium Molybdate solution and heat. NMR spectra were recorded with a Bruker Avance 400 (400 MHz) or Bruker Avance III (300 MHz). Substances were dissolved in CDCl_3_ and chemical shifts (ppm) were referenced to CHCl_3_/tetramethyl silane. Coupling constants (*J*) are given in Hz. After reaction steps solvents were evaporated with rotary evaporator (RV8 IKA, KNF SC 920) under vacuum. To purify substances, flash chromatography was performed (Interchim puriFlash 5.020 with Interchim PF-15SIHP-F0040 or PF-50SIHP-F0040 columns). Purity of reaction products was determined *via* HPLC (Varian ProStar) and ELS detection (Sedere Sedex 80). Mobile phases contained water (0.05% formic acid) and methanol (0.05% formic acid) as gradients. Stationary phase was ReproSil-Pur 120 C_18_-AQ, 5 µm, 75x3 mm (Dr. Maisch). High resolution mass spectra were recorded with a Bruker maXis 4G ESI-TOF from Daltonik [JL1], using ESI^+^ mode with following settings: Capillary voltage 4.5 kV, source temperature 200°C, gas flow 6 L/min, nebulizer gas pressure 1.2 bar, end plate offset – 0.5 kV and an m/z range of 100 to 1350. Detailed synthesis and reaction procedure can be found in SI.

### 2.2 Stability in whole blood, U937 and RPMI

Human blood products used in the *in vitro* assays (for cell stimulation and stability) were obtained from the center for transfusion medicine in Tübingen, Germany (Zentrum für Klinische Transfusionmedizin Tübingen GmbH, (ethical approval number ZKT-FoPro202106-2305-01). Test compounds (1 µM) in either culture medium (RPMI-1640 medium containing 10% fetal bovine serum, 60 mg/l Penicillin G sodium salt and 100 mg/l Streptomycin sulfate (all Biowest)), human blood (diluted 1:1 with culture medium) or a suspension of 5x10^6^ cells/ml U937 in culture medium were incubated at 37°C, 450 rpm on a shaking incubator. At the indicated time points 50 µL of blood, cell suspension or medium were collected and prepared for HPLC-MS/MS-Analysis as detailed below.

### 2.3 HPLC-MS/MS

All samples were extracted with 3 or 6 volumes acetonitrile containing terbuthylazine as an internal standard (ACN) relative to either sample weight or volume. Liquid samples (plasma, culture medium, cell suspensions in stability experiments) were diluted in either 3 (culture medium, cell suspensions) or 6 (plasma) volumes ACN, pellet and blood samples were extracted by addition of ACN followed by sonication for 5 min. Organ samples were digested with 0.5 µg/mg Proteinase K (Genaxxon) for 1 h at 50°C before being homogenized using a Fastprep FP-24 5G instrument (MP-Biomedicals). Homogenates were diluted with 6 volumes ACN and homogenized again. All extracts were cleared by centrifugation at ~20.000xg for 10 min at 4°C.

Quantification of analytes was performed on an Agilent 1260/1290 Infinity system fitted with an Agilent C18 Poroshell 120 column (4.6 x 50 mm, 2.7 µm) coupled to a triple quadrupole Sciex API 4000 MS/MS detector. The mobile phase was composed of water containing 0.1% formic acid (eluent A) and acetonitrile containing 0.1% formic acid (eluent B). Gradient used was: 5% B for 0.5 min, to 100% B in 4.5 min, 100% B for 2 min, to 5% B in 0.5 min, 5% for 2.5 min. MS detection parameters are listed in SI **Table S1**.

### 2.4 TLR7/8 SEAP reporter assay (HEK blue)

HEK blue hTLR7 or hTLR8 reporter cells (Invivogen) were cultivated in DMEM High Glucose (Biowest) according to the manufacturer’s instructions. Cells were treated with test compounds and controls at various concentrations in serum-free DMEM and incubated at 37°C, 5% CO_2_ for 24 h before supernatants were collected.

Relative secreted embryonic alkaline phosphatase (SEAP) activity in the supernatants was determined by quantification of para-Nitrophenyl Phosphate (pNPP)-turnover. Supernatants were diluted 10-fold in a solution containing 1 mM MgCl_2_, 1 M diethanolamine and 1 mg/mL pNPP and incubated at RT for 15 min before the reaction was stopped by the addition of 0.25 volumes of 1 M NaOH. Absorbance was measured at 405 nm on a Versamax microplate reader (Molecular Devices) and normalized to the mean of >5 solvent controls.

### 2.5 Viability assay and live-dead staining (MTT and dye exclusion)

3-(4,5-dimethylthiazol-2-yl)-2,5-diphenyltetrazolium bromide (MTT) turnover was used to identify potential compound effects on cell metabolism and indirectly assay changes in cell number or viability. HEK Blue reporter cells were cultured and exposed to compounds as described above and 20 µl supernatant were collected for SEAP activity assays. U937 monocyte-like cells were cultured in RPMI-1640 containing 10% FBS, differentiated by addition of 100 nM PMA for 2 days and exposed to compounds at varying concentrations or solvent for 2 days. MTT dissolved in PBS was added to the cells to a final concentration of 1 mg/ml. Cells were then incubated at culture conditions for 1 h. Supernatants were removed after centrifugation at 400xg for 5 min and the formed formazan dye was dissolved in DMSO. Absorbance was measured at 570 nm and readings were normalized to solvent treated controls.

Exclusion of Helix NIR (BioLegend) was used to assay membrane integrity following compound treatment. Culture conditions for U937 were as described above, undifferentiated cells were incubated with varying concentrations of compound or with solvent. Helix NIR was added to the cells to a final concentration of 10 nM, cells were incubated at RT for 10 min and acquired on a ZE5 Cell Analyzer (Bio-Rad). The cutoff for positive staining was set to approximately the 99^th^ percentile of unstained cells.

### 2.6 Full blood stimulation assay

Human peripheral blood of healthy donors was diluted in an equal volume of culture medium as described in 3.2, blood was treated with test compounds or controls at various concentrations and incubated at 37°C, 5% CO_2_ for 6 h. Cells were pelleted by centrifugation at 400xg for 5 min and supernatants were collected.

### 2.7 Quantification of cytokines by ELISA or cytometric bead array

Cytokine concentrations in samples were quantified either by ELISA (hTNFα, R&D Systems; hIFNα, Mabtech) or cytometric bead arrays (CBA, LegendPlex mouse anti-virus response panel, BioLegend) according to the manufacturer’s instructions. CBAs were acquired on a ZE5 Cell Analyzer (Bio-Rad) and analyzed using the LegendPlex Software Suite (Qognit/BioLegend). Absorbance of ELISA-samples was quantified using a Versamax microplate reader (Molecular Devices).

### 2.8 Experimental animals, sampling and compound formulation

**Experimental Animals**. All animal experiments were carried out in accordance with German law (35/9183.81-7/SYN 06/20). Mice, 8-18 weeks old, were purchased from Janvier Laboratories and maintained in a specific-pathogen-free animal facility with chow and water *ad libitum*. After arrival mice acclimated for a minimum of 7 days.

**Formulation**. Test compounds were prepared for application in either 1% Tween 80, 9% PEG400 in ultrapure water (Biowest) (i.v., i.p., p.o. application) or 5 mM citric acid in 0.9% saline (Braun) (subcutaneous application). If compounds were administered subcutaneously, injections were carried out into the neck crease.

**Collection of Samples**. Mice were bled from the tail vein at various timepoints. Heparin (Sigma) or K_2_-EDTA (Sigma) was added to blood samples to a final coagulant concentration of 10-15 Unit or 5 mM. Plasma was generated by centrifugation at 6800xg for 8 min at 4°C and stored at -80°C until analyzed by ELISA or CBA. Animals were sacrificed after the indicated time points by CO_2_ inhalation. Heart blood and organs for compound quantification by HPLC-MS/MS were collected post mortem and stored at –20°C until extracted as described above.

## 3 Results

**Figure 1:**
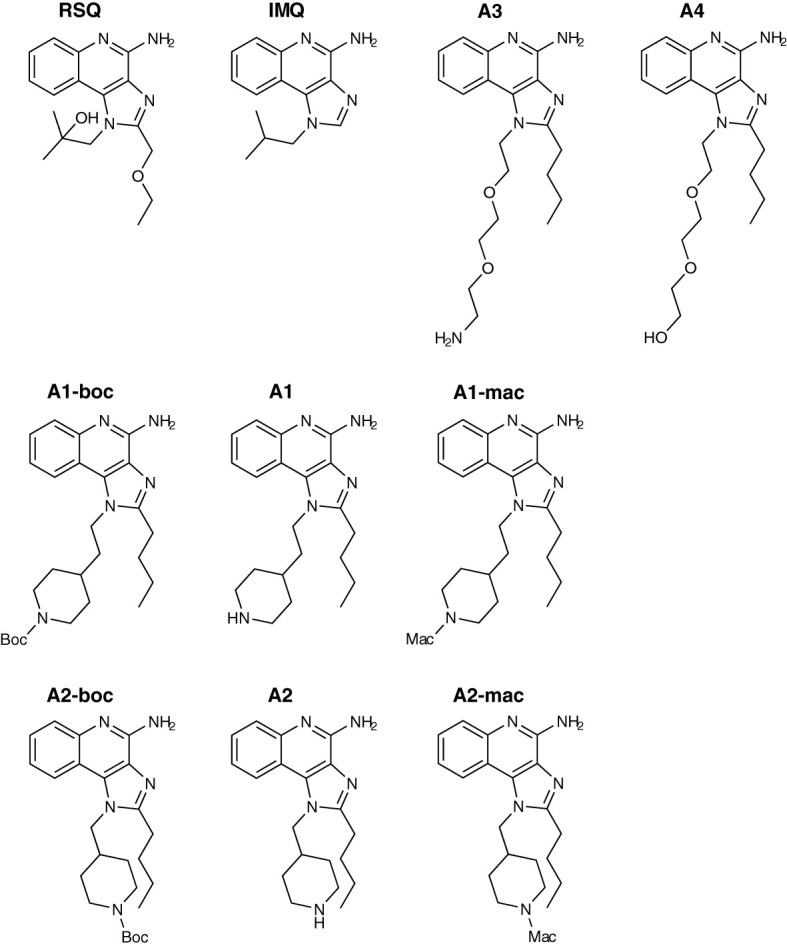
Compound structures.

**Figure 2:**
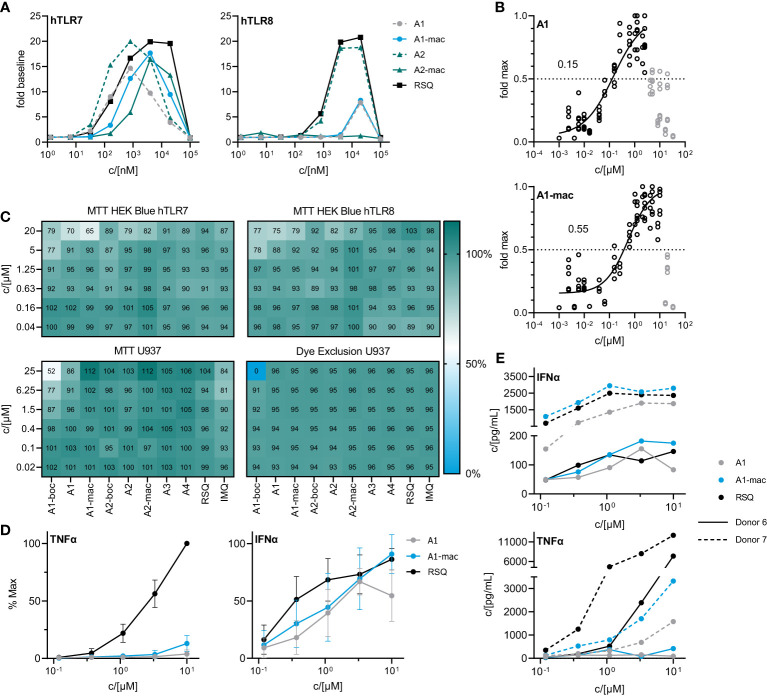
*In vitro* characterization of candidate molecules **(A)** SEAP activity in supernatants of HEK Blue reporter cells after 24 h stimulation with varying concentrations of test compounds relative to solvent controls. **(B)** Dose response of A1 and A1-mac in HEK Blue hTLR7. Circles represent normalized replicate measurements pooled from 3 experiments. Lines represent non-linear functions fit to the data to calculate compound-specific EC50; values in grey have been excluded before fitting. **(C)** Percent MTT conversion of compound-treated HEK-Blue reporter cells (top panels) or PMA-differentiated U937 relative to solvent controls OR percent of undifferentiated U937 excluding Helix NIR dye. **(D)** TNFα/IFNα in supernatants of human blood stimulated with A1, A1-mac or RSQ for 6 h, n=8, concentrations normalized to the maximum concentration of each cytokine for a given donor, data presented as mean ± 95% CI. **(E)** TNFα/IFNα in supernatant for two individual donors.

Activity of the new compounds (**Figure 1**) on TLR7/8 was confirmed using the commercially available HEK-Blue reporter system. Corresponding to the dual specificity of structurally similar compounds described by others (1, 2), HEK-Blue hTLR7 and hTLR8 were used to assess relative potency on each receptor relative to Resiquimod (RSQ) as a reference compound. Results of the initial screening are in **Table 1**; **Figure 2A**. While all structural variants retained TLR7-agonism, activation of hTLR8 was reduced for compounds linked to a macrolide, carrying a protective group or a longer spacer between the aromatic polycycle and the piperidine as in A1. Maximal induction of SEAP varied between compounds and was generally highest for RSQ in repeated experiments, while other compounds either reached a lower maximum and subsequent decrease in signal at lower concentrations or became insoluble under the conditions used for the assay before reaching a plateau. Average maximal activity in HEK-Blue hTLR7 relative to RSQ was 58% for A1, 55% for A1-boc and 41% for A1-mac. The methyl-piperidine variants reached a higher maximal induction (A2-boc 86%, A2 81%, A2-mac 63%). SEAP secretion from HEK-Blue hTLR-8 was close to baseline for A1 as well as the protected or macrolide-bound variants A1-boc, A1-mac, A2-boc and A2-mac, with the former two showing low induction at very high concentrations without reaching a plateau at sub-toxic concentrations. A2 (57%), A3 (36%) and A4 (18%) retained activity, although at a lower maximal induction than that of RSQ.

**Table 1:** EC_50_ values for compounds in HEK-blue human TLR7 and human TLR8 receptor assay (expressed as 95% CI, ND noted for no calculation of curve fit possible (adj. r² < 0.8)) and average maximal SEAP secretion observed for a given compound relative to the maximum secretion observed in the assay.

Compound	TLR7 EC_50_ [µM]	TLR7 activity rel. to assay max	TLR8 EC_50_ [µM]	TLR8 activity rel. to assay max
RSQ	0.47 to 0.77	95% ± 5%	2.9 to 3.6	96% ± 4%
IMQ	5.2 to 8.3	35% ± 7%	ND	3% ± 1%
A1-boc	0.39 to 0.69	55% ± 7%	ND	4% ± 2%
A1	0.096 to 0.22	58% ± 16%	ND	11% ± 8%
A1-mac	0.40 to 0.74	41% ± 8%	ND	5% ± 3%
A2-boc	0.23 to 0.47	86% ± 9%	ND	4% ± 1%
A2	0.075 to 0.12	81% ± 13%	2.8 to 3.1	57% ± 9%
A2-mac	1.5 to 2.0	63% ± 9%	ND	4% ± 1%
A3	1.0 to 1.5	62% ± 9%	8.2 to 8.5	36% ± 4%
A4	1.5 to 2.1	84% ± 5%	11 to 13	18% ± 8%

The compound specific EC50 for the HEK-Blue system is listed in **Table 1**. To obtain reliable estimates, replicate measurements of a minimum of 2 experiments were normalized to the maximal SEAP activity of a compound in a given experiment and pooled before fitting a non-linear function to the data. For all compounds, we observed a reduction in SEAP activity in the supernatants above certain compound concentrations. Data points above those concentrations were excluded before curve fitting (**Figures 2B**; **S3**, **S4**).

The decrease in signal was assumed to be caused by toxic effects above certain concentrations, as indicated by a change in cell morphology and reduced attachment to the plate surface. Sensitivity of the reporter cells to toxic effects seemed to be closely related to serum concentrations during the assay and was less apparent if higher serum concentrations were used (**Figure S5A**). To confirm this, we performed an MTT assay on the HEK cells after collection of the supernatants for SEAP quantification (**Figure 2C** upper two panels). While there was some reduction in MTT conversion at concentrations similar to those for which we observed a decrease in SEAP secretion, obvious toxicity could only be observed at 20 µM and is likely to be related to poor solubility and crystalizing of the compounds at those concentrations. Further, a reduction in MTT conversion could be observed in U937 cells and was again most apparent for the poorly soluble A1-boc, with 50% dye formation relative to solvent controls at the highest concentration of 25 µM. Imiquimod and A1 reduced signal to <90% at concentrations ≥6.25 µM. In contrast, a dye exclusion assay in U937 cells could only confirm negative effects on membrane integrity for cells treated with 25 µM A1-boc, pointing to additional compound effects on metabolism and proliferation of the cells as opposed to cell death.

A1 and A1-mac were selected for further studies based on their similar specificity and activity. Blood from 8 human donors was stimulated at varying concentrations of either test compounds or reference for 6 hours. Secretion of key cytokines was quantified by ELISA. IFNα and TNFα were selected as indicators for either NFκB- or IRF3/7-mediated signaling following stimulation. Maximal supernatant concentrations of both cytokines varied considerably between donors (**Figure 2E**; **Figure S5B**). When normalized to the maximal observed concentration for a given donor, relative IFNα induction was robust and similar for both A1 and A1-mac as well as the reference, while the highest TNFα concentrations were almost exclusively measured in supernatants of RSQ-treated samples (**Figure 2D**). This is consistent with the reduced affinity to TLR8 apparent in the reporter assay (**Figure 2A**). IFNα response for a given concentration was comparable for all compounds, in contrast to the lower activity of A1/A1-mac in the HEK-blue system. However, overexpression of a given TLR and signal transduction exclusively *via* NFκB instead of IRF3/7 make the HEK system useful to estimate affinity to a given receptor but might not reflect a more complex system with multiple adapter molecules involved in a primary immune cell.

**Figure 3:**
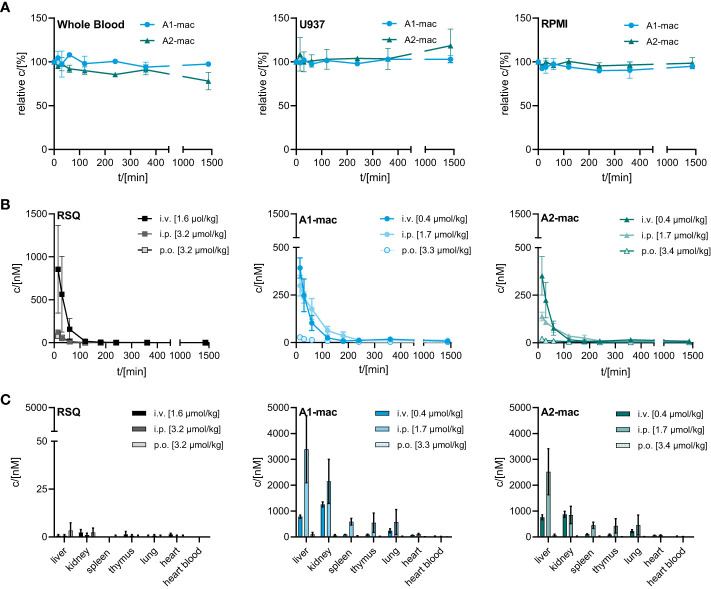
Stability and bioavailability of macrolide conjugates. **(A)** Stability of A1-mac and A2-mac was measured in human blood, U937 monocytes and RPMI medium over 24 h. **(B, C)** Concentration of RSQ, A1-mac and A2-mac in peripheral blood and organs was assessed *via* HLPC-MS after i.v., i.p. and p.o. compound administration in 10-week-old, female C57BL/6 mice (n=3 mice per group). Compounds were administered in cassettes (i.v. application: 1.6 µmol/kg RSQ, 0.4 µmol/kg A1-mac, 0.4 µmol/kg A2-mac; i.p. application: 3.2 µmol/kg RSQ, 1.7 µmol/kg A1-mac, 1.7 µmol/kg A2-mac, p.o. application: 3.2 µmol/kg RSQ, 3.3 µmol/kg A1-mac, 3.3 µmol/kg A2-mac). **(B)** Peripheral blood was collected 15, 30 60, 120, 180, 240, 360 and 1440 min after compound administration. **(C)** Organs were sampled 1440 min after compound administration. **(A–C)** Data are presented as mean ± SD.

One of the issues we addressed by coupling a TLR-activating structure to a macrolide was poor bioavailability of available TLR-agonists and the resulting limitations in possible routes for systemic treatment. We confirmed the stability of our macrolide conjugates in biological systems *in vitro*. In whole blood and cell based (U937) assays, compounds A1-mac and A2-mac were stable (**Figure 3A**) over 24 h. We next sought to investigate whether stable coupling to a carrier known for good tissue penetration and -distribution would translate to more favorable pharmacokinetics *in vivo*. To this end we compared bioavailability following intravenous (i.v.), oral (p.o.) or intraperitoneal (i.p.) application with cassettes containing A1-mac, A2-mac and RSQ. Blood samples taken from the tail vein at various times and organ samples collected terminally were then analyzed for compound concentrations by HPLC-MS/MS. Doses of compounds were selected based on known tolerance of TLR agonists per route and restricted by detection limits of analytical methods (i.v. 0.5 mg/kg; i.p. 2 mg/kg; p.o. 2 mg/kg). As expected, i.v. application (i.v. 0.5 mg/kg) showed highest blood concentrations for all substances (c_max_: RSQ 854 nM; A1-mac 392 nM; A2-mac 352 nM 15 min after application) but also showed fast elimination (baseline level after 120 min) (**Figure 3B**) and low tissue distribution (**Figure 3C**). Surprisingly, the oral availability of macrolide-bound TLR agonists was lower than expected and on the same level as RSQ (c_max_: RSQ 41 nM; A1-mac 28 nM, A2-mac 22 nM 15 min after application). This observation was unexpected, as macrolide-based substances usually possess good oral availability. This is generally accompanied by good systemic distribution and accumulation in tissues (53, 54, 56) and the relatively low plasma concentrations in this case may also reflect retention in the gut epithelium, which has been observed for other similar conjugates (53). In contrast, i.p. administration showed a distribution more similar to other macrolide-conjugates described previously by us (53). Like i.v. treatment, i.p. (2 mg/kg), had rapid partition to blood (c_max_: RSQ 123 nM; A1-mac 299 nM; A2-mac 138 nM 15 min after application) and high concentrations of compounds A1-mac and A2-mac in tissue with high levels in the liver (RSQ < LOD; A1-mac 3390 nM; A2-mac 2522 nM) and kidney (RSQ < LOD; A1-mac 586 nM; A2-mac 449 nM). Given the low levels following oral application and the risk that it may stimulate the gut excessively, the oral route was not used in subsequent *in vivo* studies.

**Figure 4:**
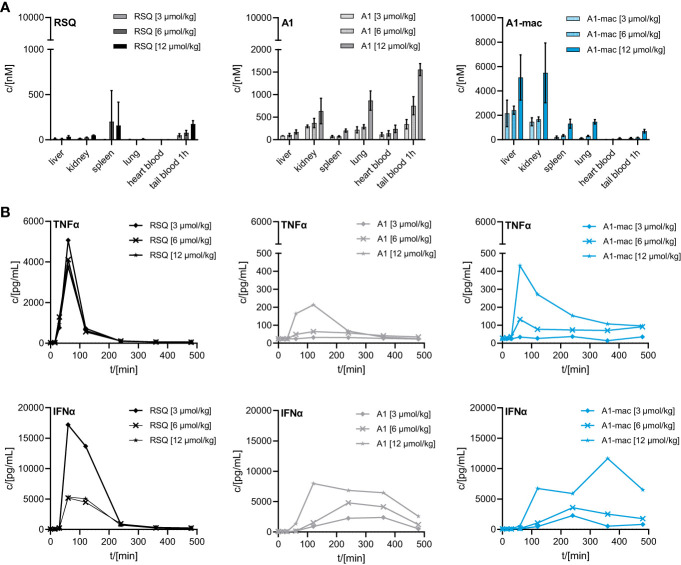
Concentration of RSQ, A1 and A1-mac in organs and cytokine profile in peripheral plasma over time after 3, 6 and 12 µmol/kg s.c. compound administration in 8-week-old, female C57BL/6 mice (n=3 mice per group). **(A)** Organs were sampled 8 h after treatment and compound concentration was determined *via* HPLC-MS/MS. Data are presented as mean ± SD. **(B)** Cytokine levels in tail plasma over time were determined *via* cytometric bead array. At each sampling timepoint the plasma of mice in one treatment group was pooled.

We then compared the activity of A1 and A1-mac *in vivo*. Since receptor engagement and activities *in vitro* were very similar for A1 and A1-mac, we hoped to be able to identify changes in activity directly related to the macrolide carrier. Compounds were applied subcutaneously at 3, 6 or 12 µmol/kg. Plasma samples taken at various times before and after treatment were analyzed for cytokine concentrations. Organs, terminal heart blood and peripheral blood 1 h post-treatment were analyzed by HPLC-MS/MS. As in the last study (**Figures 3B, C**), high concentrations of A1-mac were found in liver (5099 nM for 12 µmol/kg) and kidney (5481 nM for 12 µmol/kg) 8 h after treatment (**Figure 4A**). This was not found for RSQ (33 nM in liver and 51 nM in kidney for 12 µmol/kg) and A1 (173 nM in liver and 636 nM in kidney for 12 µmol/kg). Levels of A1 were dose dependent for all organs, with lung (868 nM for 12 µmol/kg) and tail blood high after 1 h (1557 nM for 12 µmol/kg). Concentrations measured for RSQ were generally lower with spleen being (202 nM for 6 µmol/kg and 158 nM for 12 µmol/kg) the highest of the organs analyzed.

These pharmacokinetic data confirm the known effects of macrolides on half-life and volume of distribution, are in line with our previous study (**Figure 3**) and show that s.c. is a suitable application route. For A1, the higher concentrations across all tissues point to better overall penetration and stability when compared to RSQ, further supported by higher concentrations of A1 in peripheral blood 1 h post application (A1: 1557 nM, A1-mac: 696 nM, RSQ: 175 nM, for 12 µmol/kg). Whole blood was analyzed in these studies to take account of material portioned to cells.

Similar to the blood stimulation assays described earlier (**Figure 2D**), the induction of pro-inflammatory cytokines and IFNα was clearly different between groups receiving RSQ and either A1 or A1-mac (**Figure 4B**). While RSQ treatment resulted in a sharp increase in TNFα and IFNα plasma levels, peaking 90 min post treatment and falling close to baseline after 240 min, release kinetics were generally slower in the groups receiving A1 or A1-mac. Mice treated with A1-mac had a lower TNFα peak at 90 min while in A1-treated mice it was at 120 min. IFNα levels stayed elevated over the 8 h period of the study in A1 and A1-mac treated groups.

Most striking was that in RSQ-treated groups, the peak TNFα concentrations were over 10 times higher than in A1 or A1-mac groups. The area under the curve calculated from the TNFα plasma values of mice receiving the lowest dose of 3 µmol/kg RSQ was about 4 times larger than the area calculated for any A1 or A1-mac treated group. In contrast to this, the AUCs for IFNα were similar between groups (**Table 2** top section).

**Table 2:** AUC of TNFα and IFNα measured in peripheral plasma over an 8 h period after application of equimolar doses (top section) or doses adjusted to the activity of the compounds (bottom section) in female C57BL/6.

	TNFα AUC [pg/ml∙min]						IFNα AUC [pg/ml∙min]						INFα AUC / TNFα AUC					
Dose [µmol/kg]	0.1	0.3	1	3	6	12	0.1	0.3	1	3	6	12	0.1	0.3	1	3	6	12
A1				6.5E+03	1.7E+04	3.5E+04				6.6E+05	1.3E+06	2.5E+06				77	72	102
A1-mac				6.9E+03	2.4E+04	7.6E+04				4.2E+05	9.2E+05	3.1E+06				39	41	60
RSQ				3.3E+05	2.8E+05	2.6E+05				2.2E+06	7.8E+05	8.0E+05				3	3	7
																		
A1			9.7E+03	1.1E+04	2.0E+04	4.4E+04			7.0E+04	4.9E+05	1.1E+06	1.9E+06			7	44	58	44
A1-mac			7.2E+03	1.4E+04	3.8E+04	6.5E+04			3.3E+04	3.0E+05	1.6E+06	1.9E+06			5	22	42	30
RSQ	1.7E+04	5.0E+04	1.2E+05	1.5E+05			1.1E+05	5.6E+05	1.1E+06	9.7E+05			6	11	9	6		

Plasma of 3 individual animals per group was pooled and analyzed via CBA. Values in the right section show AUCs of IFNα normalized to the corresponding AUC of TNFα for a given compound and dose.

**Figure 5:**
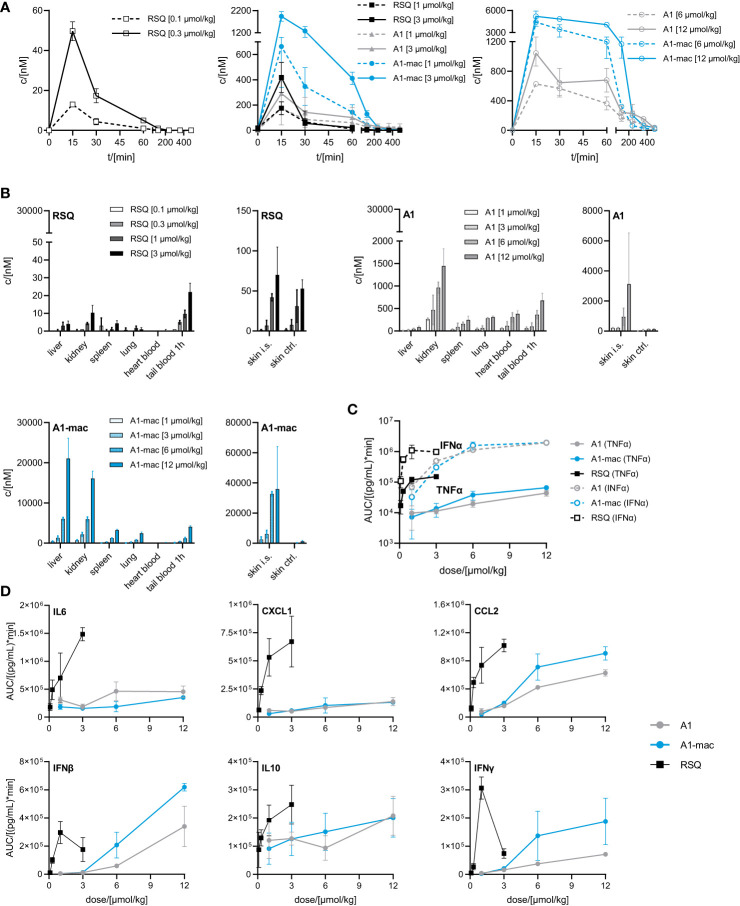
Pharmacokinetics and induction of TNFα, IFNα by A1 and A1-mac and RSQ. **(A–D)** Female, 18-week-old, C57BL/6 mice were treated s.c. with either 0.1, 0.3, 1, 3 µmol/kg RSQ, or 1, 3, 6 or 12 µmol/kg A1 or A1-mac (n=3 mice per group). Compound concentration in **(A)** peripheral blood collected *via* tail bleeding before, 15, 30 60, 120, 240, 360 and 480 min after s.c. compound application and **(B)** organs collected after 8 h was assessed *via* HPLC-MS/MS. **(A, B)** Data are presented as mean ± SD. **(C)** Levels of TNFα and IFNα as well as **(D)** IL6, CXCL1, CCL2, IFNβ, IFNγ and IL10 in tail plasma over time were determined *via* cytometric bead array. Area under the curve (AUC) of each cytokine was plotted against compound concentration. Data are represented as mean ± 95% confidence interval.

While we anticipated different release kinetics based on the differences in pharmacokinetics described earlier, the different cytokine release patterns were unexpected. Preference for TLR7 over TLR8 should not have an impact in murine systems (in which activity of RSQ is thought to be dependent on TLR7 under normal circumstances (62, 63)) and the release of TNFα as well as IFNα and other cytokines (**Figure S6**) were highest in the RSQ group receiving the lowest dose. We suspected this to be due to a saturation effect and possibly overshooting feedback mechanisms. The idea of negative feedback potentially decreasing the secretion of Type I IFN in RSQ treated animals after a short burst is supported by the higher IL10 levels observed only in those animals (**Figure S6** bottom panels). In this case, differences in cytokine secretion could be explained simply by the higher potency of RSQ compared to A1/A1-mac. To rule out differences in potency as the reason for the varying cytokine profiles, we reduced RSQ doses to 0.1, 0.3, 1 and 3 µmol/kg and added 1 µmol/kg as an additional dose for A1 and A1-mac in a follow-up study. The doses were chosen so the lowest dose for a given compound would be at the threshold of detectable activity while reflecting the differences in maximal TLR7-activation we originally observed in the HEK reporter assay. When comparing the AUC in this study, we found a dose ratio of roughly 6-10 times the molar dose of RSQ leading to comparable amounts of IFNα in A1 and A1-mac treated groups, while 30-40 times the molar dose were necessary to induce similar levels of TNFα. More specifically, we could not find a dose for which RSQ would induce similarly high levels of type I Interferon without also leading to much higher release of TNFα than A1 and A1-mac (**Figure 5C**; **Table 2** bottom section). This is not limited to a reduction of TNFα-secretion relative to Type I IFN but a similar pattern can be observed for other NFκB-induced cytokines (**Figure 5D**).

We conclude from this that the specific induction of high levels of Type I IFN is a characteristic feature of A1 and A1-mac and cannot be reproduced by any dose of RSQ. These characteristics might be related to the different stability and pharmacokinetic profile when compared to RSQ, to varying receptor specificity or to differences in subcellular partitioning of the compounds.

Distribution of compounds to different organs was similar in pattern but varied in concentration when compared to the previous study (**Figures 5B**, **4A**). Taking the different dose ranges into account, A1 and A1-mac reach higher concentrations in tissues than RSQ 8 h post compound application (in kidney at 3 µmol/kg RSQ: 10 nM A1: 469 nM A1-mac 2176 nM and in liver RSQ: <LLOQ A1: 45 nM A1-mac 6030 nM). Compound levels in peripheral plasma over time were analyzed in this study. We found that not only the macrolide conjugate but also the free agonist A1 was detectable in plasma over a longer period of time when compared to RSQ. This may, in part, be due to higher stability as well as retention at and slower release from the injection site for A1 and A1-mac (**Figure 5B**, narrow panels).

Since we could not attribute our observations to dose alone and there are specific cases in which murine TLR8 is reported to be activated (64), we added Imiquimod as the prototypical TLR7-agonist to act as an additional reference in our next study. Imiquimod itself is not solely reliant on TLR7 signaling to trigger its pro-inflammatory effects, being also an inhibitor of adenosine receptors (65). However, it is inactive on TLR8 and more similar to A1 and A1-mac in that regard. To account for the lower potency of Imiquimod when compared to the other compounds, we used a dose corresponding to the ~10-fold difference in potency relative to A1-mac indicated by the reporter assay detailed earlier. We further chose doses for A1, A1-mac and RSQ based on those which resulted in similar IFNα-AUC in the previous study and modified the protocol to include three consecutive daily treatments to assess the effect of repeated applications on pharmacokinetics (induced metabolism, accumulation) and cytokine induction.

**Figure 6:**
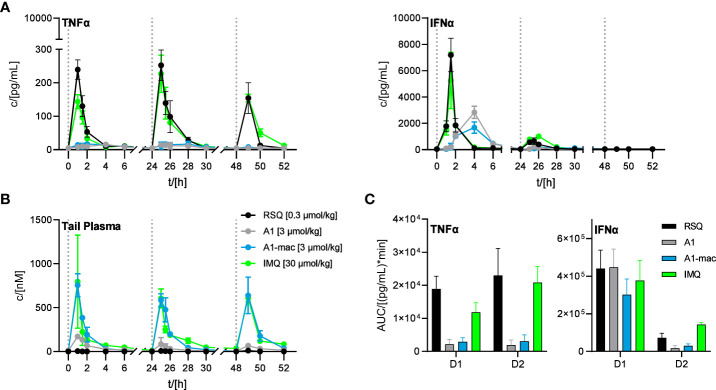
Effect of repeated applications of A1, A1-mac, RSQ and IMQ. Dotted lines indicate the time of repeated compound applications. **(A)** Cytokine levels in pooled tail plasma of mice from individual treatment groups (n=3 per pool and time point), error bars represent range of two replicate measurements. **(B)** Peripheral blood was collected from the tail vein before, 15, 30 60, 120, 240, 360 and 480 min after s.c. treatment. Compound concentration in peripheral plasma was analyzed by HPLC-MS/MS, data presented as mean ± SD. **(C)** AUC of TNFα/IFNα in peripheral plasma calculated from the data in **(A)** for days 1 and 2 of treatment, data represent calculated AUC ± 95% CI.

Organ concentrations (**Figure S7**; collected after 4, 28 and 52 h) were similar to previous studies, with highest concentrations in liver (RSQ: <LLOQ IMQ: 630 nM A1: 63 nM A1-mac: 2617 nM after 4 h; RSQ: <LLOQ IMQ: 824 nM A1: 82 nM A1-mac: 4461 nM after 28 h; RSQ: <LLOQ IMQ: 645 nM A1: 94 nM A1-mac: 6352 nM after 52 h). Interestingly, measured brain tissues showed baseline or close to baseline levels for RSQ (most likely at least partly due to the comparatively low dose), A1 and A1-mac, whilst IMQ was detected with increasing concentrations over the course of the study (32 nM after 4 h; 135 nM after 28 h, 177 nM after 52 h), in line with earlier publications and our observations connecting IMQ brain concentrations with systemic inflammatory responses (66, 67). While there were some minor deviations in compound plasma levels between the first and consecutive treatments (all peaked 60 min after each application; day 1: RSQ <LLOQ IMQ 796 nM, A1 171 nM, A1-mac 755 nM; day 2: RSQ <LLOQ, IMQ 606 nM, A1 85 nM, A1-mac 585 nM; day 3: RSQ <LLOQ, IMQ 608 nM, A1 66 nM, A1-mac 639 nM; **Figure 6B**), the most prominent effect of repeated doses is a decline in Interferon-secretion after the first treatment. Secretion of TNFα was fairly similar after each of the repeated treatments and remained low for all treatments with A1 and A1-mac in comparison with IMQ and RSQ (**Figure 6C** shows days 1 and 2). IFNα induction was reduced after the second treatment for all compounds and plasma levels on the third day generally remained below the limit of detection. The decrease in INFα levels on the second day was more pronounced for A1 and A1-mac, both being at the limit of detection for type I Interferon concentrations in plasma after the second treatment (**Figure 6A**).

The induction of IFNα and TNFα after treatment with RSQ or IMQ were very similar in their kinetics as well as the ratio of both cytokines. A1- and A1-mac-treated animals showed a delayed and more sustained induction of IFNα and little TNFα in peripheral plasma after the first treatment, as in previous studies.

## 4 Conclusion

The similarities between IMQ and RSQ in cytokine induction make differences in receptor specificity an unlikely explanation for the divergent cytokine profile induced by A1/A1-mac. We hypothesize that these observations are due to either PK and specifically release kinetics from the injection site or possibly partitioning to specific cellular compartments. The differences in PK are clear from the data reported here.

Additionally, we consider the option that subcellular location may be relevant in “polarizing” TLR7-mediated signaling based on: firstly, the observations made by others demonstrating the outcome of TLR9 activation is dependent on the cellular compartment in which activation occurs (28–31); and, secondly, the considerable overlap between adapter molecules employed by TLR7 and 9 to either activate NFκB or cause phosphorylation of IRF7, particularly TRAF6 or TRAF3. Thirdly, our own observations that a structurally similar fluorescent tool compound consisting of a macrolide core conjugated to a coumarin dye accumulated in endosomal compartments (Laux et al., in review) which could be organelles relevant to IRF7 activation as well as the research of others demonstrating endosomal uptake of both macrolides and imidazoquinolines (55, 60). In conclusion, we consider the possibility that preferential uptake of our compounds in those organelles causes their characteristic cytokine induction, although preferential partitioning to specific endosomes needs to be explicitly demonstrated.

Irrespective of the exact mechanism causing them, these data suggest that the compounds have profound and distinct properties and biological activities relative to well-known compounds like RSQ and IMQ. The new class differs from previous compounds in stability, distribution, spectrum and duration of action. The conserved activity of the macrolide conjugate A1-mac indicates that the compounds tolerate large bulky substituents at the linkage position and are also suitable for linkage to other macromolecules This makes them potentially useful reagents for addition of immune stimulatory properties to other compounds and agents such as polymers, proteins and antibodies.

The absence of a strong TNFα signal could increase the tolerability of the compounds in clinical use. It remains to be seen whether this is advantageous for applications in oncology. However, a variety of tumors appear to benefit from high TNFα levels and this aspect may require more nuanced investigation.

This cytokine profile with its emphasis on IFNα may potentially suit applications in treatment of viral infections. The potency and specificity of the compounds as well as their induction of patient-specific quantities of type I IFN could make them suitable as an alternative to treatment with a fixed dose of recombinant IFNα. Nevertheless, the loss of the IFNα response on successive application may indicate a risk of receptor saturation and immune exhaustion. The impact of time between treatments on this effect has been demonstrated for RSQ in the past (68) and careful attention is required to define a suitable dosing interval before application in a clinical setting.

## Data availability statement

The original contributions presented in the study are included in the article/**Supplementary Materials**. Further inquiries can be directed to the corresponding author.

## Ethics statement

The studies involving human participants were reviewed and approved by the Ethik-Kommission, Medizinische Fakultät, Universitätsklinikum Tübingen. Written informed consent for participation was not required for this study in accordance with the national legislation and the institutional requirements. The animal studies were reviewed and approved by the Regierungspräsidium Tübingen under application no. 35/9183.81-7/SYN 06/20.

## Author contributions

SS and JHG were involved in compound synthesis and characterization. SG, NS, TF, SS, and MK designed and carried out *in vitro* experiments and processed samples from *in vivo* studies. SG, TW, MB, and MK designed and carried out *in vivo* experiments. AS, JG, SS, and MK processed samples for and data from HPLC-MS/MS measurements. MK, SS, SG, and MB wrote the manuscript. MB and SL supervised the project and provided funding. All authors proof-read the manuscript. All authors contributed to the article and approved the submitted version.
