# Supplementary material for: Imidazoquinolines with improved pharmacokinetic properties induce a high IFNα to TNFα ratio in vitro and in vivo
Source: Front Immunol. 2023 Jun 20;14:1168252. doi: 10.3389/fimmu.2023.1168252 (PMC10319141; doi:10.3389/fimmu.2023.1168252)
Supplement: Supplementary file 1 [file Presentation_1.pdf]

1. Synthesis

Compound S1.2 3-nitroquinoline-2,4-diol, ^{1}H NMR (300 MHz, DMSO) δ 11.98 (s, 1H), 11.36 (s, 1H), 8.01 (d, *J* = 8.1 Hz, 1H), 7.61 (t, *J* = 7.7 Hz, 1H), 7.32 (d, *J* = 8.2 Hz, 1H), 7.24 (t, *J* = 7.6 Hz, 1H), ^{13}C NMR (75 MHz, DMSO) δ 156.49, 155.91, 138.24, 133.39, 133.14, 127.29, 124.69, 124.46, 122.56, 122.33, 116.11, 115.86, 114.07, yellow solid, yield 37.1 g (72.6%), MS $(M+H)^+$ calcd *m/z* 207.04, found *m/z* 207.20. Synthesis of intermediate S1.2 was done following Shukla *et al.* (J. Med. Chem, 2010). Briefly, quinoline-2,4-diol (TCI D1753, 40 g, 0.248 mol) was dissolved in nitric acid (53%, 300 mL) and stirred at RT for 10 min. The mixture was then heated to 75 °C for 15 min. After cool down to RT the solution was poured into ice/water mixture to form solid yellow precipitate (S1.2). Precipitate was filtered off and dried to yield 37.1 g. and used without further purification.

Compound S1.3 2,4-dichloro-3-nitroquinoline, ^{1}H NMR (300 MHz, $CDCl_3$) δ 8.16 (d, *J* = 8.4 Hz, 1H), 8.00 (d, *J* = 8.4 Hz, 1H), 7.88 (t, *J* = 7.6 Hz, 1H), 7.74 (t, *J* = 7.6 Hz, 1H), ^{13}C NMR (75 MHz, $CDCl_3$) δ 146.50, 139.59, 135.62, 133.39, 129.72, 129.05, 125.24, 125.04, 124.33, 77.58, 77.16, 76.74, 49.55, 49.27, 48.98, greyish solid, yield 10.7 g (96.9%). Synthesis of intermediate S1.3 was done following Shukla *et al.* (J. Med. Chem, 2010). Briefly, compound S1.2 (9.4 g, 45.6 mmol) was dissolved in 60 mL of phenylphosphonyl dichloride (VWR A13777) and heated to 135 °C for 3 h. Solution was transferred into ice/water mixture and stirred vigorously. Formed precipitate was filtered off and dried to yield 10.7 g of S1.3 and used without further purification.

Compound S1.4-A1 tert-butyl 4-(2-((2-chloro-3-nitroquinolin-4-yl)amino)ethyl)piperidine-1-carboxylate, white solid, yield 3.1 g (57.6%), MS $(M+H)^+$ calcd *m/z* 435.18, found *m/z* 435.13. Compound S1.3 (3 g, 12.4 mmol) was dissolved in anhydrous dichloromethane with triethylamine (2.59 mL, 1.5 eq). To this solution tert-butyl 4-(2-aminoethyl)piperidine-1-carboxylate (ChemPur BD31964-5, 3.4 g, 14.9 mmol) was added. The reaction mixture was heated to 45°C for 45 min. Reaction progress was monitored via TLC (ethyl acetate/acetone/cyclohexane 2:1:3 with 0.5% triethylamine). Solvents were removed under vacuum, residue washed with water and dried. Crude product was then purified via column chromatography on silica gel (400 g) with a mixture of cyclohexane and ethyl acetate, eluted with a with a gradient of solvents to obtain 3.1 g of S1.4-A1.

Compound S1.5-A1 tert-butyl 4-(2-((3-amino-2-chloroquinolin-4-yl)amino)ethyl)piperidine-1-carboxylate, yellow solid, yield 1.06 g (>99%), MS $(M+H)^+$ calcd *m/z* 405.21, found *m/z* 405.27. Compound S1.4-A1 (1.15 g, 2.65 mmol) was dissolved in water/ethyl acetate (1:1). Subsequently 3.34 g of sodium dithionite (Fisher S/3800/63) was added and solution was heated to 55 °C. After 2 min MS analysis showed full conversion to product. Product was extracted with liquid-liquid extraction (three times with dichloromethane). Organic phases were combined, dried with sodium sulphate and solvent was evaporated under vacuum to yield 1.06 g of S1.5-A1 and used without further purification.

Compound S1.6-A1 tert-butyl 4-(2-(2-butyl-4-chloro-1H-imidazo[4,5-c]quinolin-1-yl)ethyl)piperidine-1-carboxylate, white solid, yield 1.12 g (50%), MS $(M+H)^+$ calcd *m/z* 471.25, found *m/z* 471.40. Compound S1.5-A1 (1.94 g, 4.8 mmol) was dissolved in 5 mL trimethyl orthovalerate (VWR L20377.22) and solution was heated to 135 °C over three days (heating was stopped during nights). Reaction solution was poured into cyclohexane and formed precipitate was filtered of and dried to obtain 1.12 g of S1.6-A1 and used without further purification.

Compound S1.7-A1 tert-butyl 4-(2-(2-butyl-4-hydrazineyl-1H-imidazo[4,5-c]quinolin-1-yl)ethyl)piperidine-1-carboxylate, MS $(M+H)^+$ calcd *m/z* 467.31, found *m/z* 467.33. Compound S1.6-A1 (640 mg, 1.36 mmol) was dissolved in 25 mL of ethanol, subsequently hydrazine hydrate (Sigma-Aldrich 225819-500ML, 1.5 mL, 47 mmol) was added. Solution was refluxed overnight. MS analysis showed full conversion. Product S1.7-A1 was directly used for next reaction step

Compound S1.8-A1 (also A1-Boc) tert-butyl 4-(2-(4-amino-2-butyl-1H-imidazo[4,5-c]quinolin-1-yl)ethyl)piperidine-1-carboxylate, ^{1}H NMR (300 MHz, $CDCl_3$) δ 7.84 (dd, *J* = 13.6, 8.2 Hz, 2H), 7.48 (t, *J* = 7.5 Hz, 1H), 7.31 (t, *J* = 7.5 Hz, 1H), 5.80 (s, 2H), 4.46 – 4.35 (m, 2H), 4.15 (d, *J* = 9.9 Hz, 2H), 2.89 – 2.80 (m, 2H), 2.75 (t, *J* = 12.4 Hz, 2H), 1.93 – 1.77 (m, 5H), 1.74 (s, 1H), 1.71 – 1.59 (m, 1H), 1.51 (dt, *J* = 9.8, 4.8 Hz, 2H), 1.46 (s, 9H), 1.28 (dt, *J* = 19.7, 6.1 Hz, 2H), 1.00 (t, *J* = 7.3 Hz, 3H), ^{13}C NMR (75 MHz, $CDCl_3$) δ 154.87, 153.30, 151.26, 144.07, 133.28, 127.17, 126.84, 126.80, 122.48, 119.38, 115.31, 79.71, 43.61, 36.95, 34.31, 32.07, 30.25, 28.57, 27.24, 27.02, 22.76, 13.97, white solid, yield 294 mg (48% over two steps of synthesis), purity >99% via HPLC-ELSD, MS $(M+H)^+$ calcd *m/z* 452.30, found *m/z* 452.40. Compound S1.7-A1 dissolved in 10 mL of acetic acid and subsequently three spatula tips of Zn powder were added. Reaction progress was controlled via MS. After 48 h another spatula tip of Zn powder and 1 mL of acetic acid was added. Next day full conversion was observed. The reaction mixture was diluted with water and underlayered with dichloromethane. Mixture was vigorously stirred and potassium hydroxide added until Zn formed a soluble complex. Product was extracted with liquid-liquid extraction (three times with dichloromethane). Organic phases were combined, dried with sodium sulphate and solvent was evaporated under vacuum to obtain 632 mg of crude product. To purify substance flash chromatography was performed with a gradient of cyclohexane and isopropanol (with 0.25% triethylamine each) to obtain 294 mg of compound S1.8-A1.

Compound A1 2-butyl-1-(2-(piperidin-4-yl)ethyl)-1H-imidazo[4,5-c]quinolin-4-amine, ^{1}H NMR (400 MHz, $CDCl_3$) δ 7.94 – 7.85 (m, 1H), 7.81 (dd, *J* = 8.3, 0.8 Hz, 1H), 7.53 – 7.40 (m, 1H), 7.33 – 7.27 (m, 1H), 5.40 (d, *J* = 92.9 Hz, 1H), 4.46 – 4.35 (m, 2H), 3.14 (d, *J* = 12.1 Hz, 2H), 2.89 – 2.82 (m, 2H), 2.67 (td, *J* = 12.0, 2.0 Hz, 2H), 1.95 – 1.71 (m, 6H), 1.71 – 1.56 (m, 1H), 1.50 (dq, *J* = 14.7, 7.4 Hz, 2H), 1.32 (ddd, *J* = 15.7, 12.3, 3.9 Hz, 2H), 1.00 (t, *J* = 7.4 Hz, 3H), ^{13}C NMR (101 MHz, $CDCl_3$) δ 153.22, 151.33, 144.82, 133.32, 127.42, 126.99, 122.30, 119.49, 115.63, 46.61, 43.61, 37.56, 34.56, 33.35, 30.35, 27.28, 22.78, 13.95, yellowish white solid, yield 82 mg (>99%), purity 97.5% via HPLC-ELSD, MS $(M+H)^+$ calcd *m/z* 352.25, found *m/z* 352.67. Compound S1.8-A1 (100 mg, 0.22 mmol) was dissolved in a mixture of dichloromethane and trifluoroacetic acid (10:1, 10 mL) and stirred at RT. Reaction progress was controlled via MS. To neutralize solution sodium carbonate was added after reaction was complete. Product was extracted with liquid-liquid extraction (three times with dichloromethane). Organic phases were combined, dried with sodium sulphate and solvent was evaporated under vacuum to obtain 82 mg of product A1.

Compound S1.4-A2 tert-butyl 4-(((2-chloro-3-nitroquinolin-4-yl)amino)methyl)piperidine-1-carboxylate, white solid, yield 2.3 g (46%), MS $(M+H)^+$ calcd *m/z* 421.16, found *m/z* 421.20. Compound S1.3 (2.9 g, 12.0 mmol) was dissolved in 30 mL of anhydrous dichloromethane with triethylamine (2.5 mL, 1.5 eq). To this solution tert-butyl 4-(aminomethyl)piperidine-1-carboxylate (ChemPur 11389, 3.1 g, 14.5 mmol) was added. The reaction mixture was heated to 45 °C for 45 min. Reaction progress was monitored via TLC (ethyl acetate/acetone/cyclohexane 4:1:2 with 0.5% triethylamine). Solvents were removed under vacuum, residue washed with water and dried. Crude product was then purified via column chromatography on silica gel (300 g) with a mixture of cyclohexane, ethyl acetate and acetone, eluted with a gradient of solvents to obtain 2.3 g of S1.4-A2.

Compound S1.5-A2 tert-butyl 4-(2-((3-amino-2-chloroquinolin-4-yl)amino)ethyl)piperidine-1-carboxylate, white solid, yield 1.66 g (>99%), MS $(M+H)^+$ calcd *m/z* 391.19, found *m/z* 391.27. Compound S1.4-A1 (1.78 g, 4.24 mmol) was dissolved in water/ethyl acetate (1:1). Subsequently 5.34 g of sodium dithionite (Fisher S/3800/63) was added and solution was heated to 75 °C. After 20 min MS analysis showed full conversion to product. Product was extracted with liquid-liquid extraction (three times with dichloromethane). Organic phases were combined, dried with sodium sulphate and solvent was evaporated under vacuum to yield 1.66 g of S1.5-A1 and used without further purification.

Compound S1.6-A2 tert-butyl 4-((2-butyl-4-chloro-1H-imidazo[4,5-c]quinolin-1-yl)methyl)piperidine-1-carboxylate, ^{1}H NMR (300 MHz, $CDCl_3$) δ 8.18 – 8.03 (m, 1H), 8.02 – 7.91 (m, 1H), 7.59 (dq, *J* = 7.0, 5.5 Hz, 2H), 4.36 (d, *J* = 6.9 Hz, 2H), 4.12 (d, *J* = 10.0 Hz, 2H), 2.90 (dd, *J* = 22.0, 13.9 Hz, 2H), 2.53 (t, *J* = 12.3 Hz, 2H), 2.09 (t, *J* = 9.3 Hz, 1H), 1.87 (dt, *J* = 15.6, 7.7 Hz, 2H), 1.63 – 1.45 (m, 4H), 1.39 (d, *J* = 12.5 Hz, 9H), 1.35 – 1.23 (m, 2H), 0.95 (dd, *J* = 14.2, 6.9 Hz, 3H), ^{13}C NMR (75 MHz, $CDCl_3$) δ 156.28, 154.61, 143.95, 143.79, 134.72, 133.95, 130.33, 127.48, 126.74, 119.57, 117.60, 79.86, 77.58, 77.16, 76.74, 51.17, 37.24, 30.30, 29.70, 28.44, 27.76, 22.75, 13.88, white solid, yield 2.71 g (86%), MS $(M+H)^+$ calcd *m/z* 457.24, found *m/z* 457.40. Compound S1.5-A2 (2.69 g, 6.9 mmol) was dissolved in 10 mL of dimethylformamide. To this 5 mL trimethyl orthovalerate (VWR L20377.22) were added and solution was heated to 135 °C overnight. The next day 1 mL trimethyl orthovalerate was added and solution was heated over weekend. MS analysis showed complete reaction. Crude product was purified via column chromatography on silica gel (300 g) with a mixture of cyclohexane and diethyl ether, eluted with a gradient of solvents to obtain 2.71 g of S1.6-A2.

Compound S1.7-A2 tert-butyl 4-(2-(2-butyl-4-hydrazineyl-1H-imidazo[4,5-c]quinolin-1-yl)methyl)piperidine-1-carboxylate, MS $(M+H)^+$ calcd *m/z* 453.30, found *m/z* 453.33. Compound S1.6-A2 (2.7 g, 5.92 mmol) was dissolved in 50 mL of ethanol, subsequently hydrazine hydrate (Sigma-Aldrich 225819-500ML, 4 mL, 126 mmol) was added. Solution was refluxed over two days. MS analysis showed full conversion. Product S1.7-A2 was directly used for next reaction step.

Compound S1.8-A2 (also A2-Boc) tert-butyl 4-((4-amino-2-butyl-1H-imidazo[4,5-c]quinolin-1-yl)methyl)piperidine-1-carboxylate, ^{1}H NMR (300 MHz, $CDCl_3$) δ 7.83 (t, *J* = 7.0 Hz, 2H), 7.50 (dd, *J* = 11.3, 4.2 Hz, 1H), 7.30 (dd, *J* = 11.6, 4.6 Hz, 1H), 5.51 (s, 2H), 4.31 (d, *J* = 6.9 Hz, 2H), 4.14 (d, *J* = 8.1 Hz, 2H), 2.91 – 2.83 (m, 2H), 2.56 (t, *J* = 12.4 Hz, 3H), 2.26 – 2.04 (m, 1H), 1.86 (dt, *J* = 15.4, 7.6 Hz, 2H), 1.51 (dd, *J* = 15.1, 7.6 Hz, 3H), 1.44 (s, 9H), 1.20 (d, *J* = 6.1 Hz, 1H), 0.99 (t, *J* = 7.3 Hz, 4H), ^{13}C NMR (75 MHz, $CDCl_3$) δ 154.75, 153.92, 151.36, 144.99, 133.30, 127.58, 127.15, 127.00, 122.30, 119.50, 115.71, 79.85, 50.95, 43.54, 37.31, 30.25, 29.81, 28.54, 27.64, 25.51, 22.74, 13.99, white solid, yield 332 mg (13% over two steps of synthesis), purity 96% via HPLC-ELSD, MS $(M+H)^+$ calcd *m/z* 438.29, found *m/z* 438.37. Compound S1.7-A2 dissolved in 15 mL of acetic acid and subsequently three spatula tips of Zn powder were added. Reaction progress was controlled via MS. After 48 h acetic acid was diluted with 100 mL water and underlayered with 200 mL dichloromethane. Mixture was vigorously stirred and potassium hydroxide added until Zn formed a soluble complex. Product was extracted with liquid-liquid extraction (three times with dichloromethane). Organic phases were combined, washed with water and brine, dried with sodium sulphate and solvent was evaporated under vacuum to obtain 2.56 g of crude product. To purify substance flash chromatography was performed with a gradient of cyclohexane, dichloromethane and isopropanol (with 0.25% triethylamine each) to obtain 332 mg of compound S1.8-A2.

Compound A2 2-butyl-1-(2-(piperidin-4-yl)methyl)-1H-imidazo[4,5-c]quinolin-4-amine, ^{1}H NMR (400 MHz, $CDCl_3$) δ 7.86 (dt, *J* = 6.7, 3.3 Hz, 1H), 7.82 (dd, *J* = 8.4, 0.9 Hz, 1H), 7.52 – 7.45 (m, 1H), 7.34 – 7.27 (m, 1H), 5.56 (s, 2H), 4.30 (d, *J* = 7.1 Hz, 2H), 3.09 (d, *J* = 12.1 Hz, 2H), 2.91 – 2.85 (m, 2H), 2.50 (t, *J* = 11.2 Hz, 2H), 2.19 – 2.05 (m, 1H), 1.86 (dt, *J* = 15.5, 7.6 Hz, 2H), 1.61 (d, *J* = 11.5 Hz, 2H), 1.48 (td, *J* = 14.8, 7.3 Hz, 2H), 1.43 – 1.31 (m, 2H), 1.28 – 1.15 (m, 1H), 1.02 – 0.96 (m, 3H), ^{13}C NMR (101 MHz, $CDCl_3$) δ 154.12, 151.36, 144.81, 133.45, 127.42, 127.14, 126.97, 122.30, 119.70, 115.80, 51.44, 46.18, 37.37, 30.83, 30.25, 27.64, 22.76, 13.96, yellowish white solid, yield 127 mg (75%), purity 98.5% via HPLC-ELSD, MS $(M+H)^+$ calcd *m/z* 338.23, found *m/z* 338.56. Compound S1.8-A2 (205 mg, 0.47 mmol) was dissolved in a mixture of dichloromethane and trifluoroacetic acid (10:1, 20 mL) and stirred at RT. After 30 min reaction was completed. Solution was poured into water and neutralized with potassium hydroxide. Product was extracted with liquid-liquid extraction (three times with dichloromethane). Organic phases were combined, washed with water and brine, dried with sodium sulphate and solvent was evaporated under vacuum to obtain 127 mg of A2.

Compound S1.4-A3 tert-butyl (2-(2-(2-((2-chloro-3-nitroquinolin-4-yl)amino)ethoxy)ethoxy)ethyl)carbamate, ^{1}H NMR (300 MHz, $CDCl_3$) δ 7.89 (d, *J* = 8.1 Hz, 1H), 7.82 (d, *J* = 8.4 Hz, 1H), 7.67 (dd, *J* = 8.2, 7.1 Hz, 1H), 7.48 (t, *J* = 7.7 Hz, 1H), 6.44 (s, 1H), 4.98 (s, 1H), 3.68 (dd, *J* = 9.6, 4.6 Hz, 2H), 3.63 (s, 3H), 3.57 – 3.45 (m, 4H), 3.28 (d, *J* = 5.1 Hz, 2H), 2.11 (s, 1H), 1.37 (s, 9H), ^{13}C NMR (75 MHz, $CDCl_3$) δ 156.03, 146.15, 144.66, 142.16, 132.05, 129.32, 127.79, 126.83, 126.56, 121.52, 119.32, 79.37, 77.58, 77.16, 76.74, 70.33, 68.78, 43.74, 40.30, 28.42, 28.36, white solid, yield 1.23 g (80%), MS $(M+H)^+$ calcd *m/z* 455.17, found *m/z* 455.13. Compound S1.3 (813 mg, 3.37 mmol) was dissolved in 10 mL of anhydrous dichloromethane with triethylamine (697 µL, 1.5 eq). To another 5 mL of anhydrous dichloromethane 1 g of tert-butyl (2-(2-(2-aminoethoxy)ethoxy)ethyl)carbamate (ABCR AB165344) was added. Both solutions were combined and heated to 45 °C for 3 h. Solvents were removed under vacuum. Crude product was then purified via column chromatography on silica gel (300 g) with a mixture of cyclohexane, ethyl acetate (with 0.5% of triethylamine) eluted with a gradient of solvents to obtain 1.23 g of S1.4-A3.

Compound S1.5-A3 tert-butyl (2-(2-(2-((3-amino-2-chloroquinolin-4-yl)amino)ethoxy)ethoxy)ethyl)carbamate, white solid, yield 879 mg (76%), MS $(M+H)^+$ calcd *m/z* 425.20, found *m/z* 425.27. Compound S1.4-A3 (1.23 g) was dissolved in ethyl acetate in a closed vessel. Vessel was flushed with Ar (evaporated and flushed three times). To solution a spatula of sodium sulphate and 500 mg of Pt/C (5% Pt, Sigma-Aldrich 205931-1G). The vessel was again flushed three times with Ar. Subsequently the vessel was flushed with H_2 (Air Liquide 130415). After two days of stirring reaction was completed, solution filtered through celite and solvent removed under vacuum to yield 879 mg of S1.5-A3 and used without further purification.

Compound S1.6-A3 tert-butyl (2-(2-(2-(2-butyl-4-chloro-1H-imidazo[4,5-c]quinolin-1-yl)ethoxy)ethoxy)ethyl)carbamate, white solid, yield 7.4 g (99%), MS $(M+H)^+$ calcd *m/z* 491.24, found *m/z* 491.33. Compound S1.5-A3 (6.5 g, 15.3 mmol) was dissolved in 10 mL trimethyl orthovalerate (VWR L20377.22) and solution was heated to 135 °C over three days. Residues of trimethyl orthovalerate were removed under vacuum (approx. 80 mbar) at 100 °C to yield 7.4 g of S1.6-A3.

Compound S1.7-A3 tert-butyl (2-(2-(2-(2-butyl-4-hydrazineyl-1H-imidazo[4,5-c]quinolin-1-yl)ethoxy)ethoxy)ethyl)carbamate, MS $(M+H)^+$ calcd *m/z* 487.30, found *m/z* 487.40. Compound S1.6-A3 (7.4 g, 15.1 mmol) was dissolved in 35 mL of ethanol, subsequently hydrazine hydrate

(Sigma-Aldrich 225819-500ML, 5 mL, 158 mmol) was added. Solution was refluxed over two days. MS analysis showed full conversion. Product S1.7-A3 was directly used for next reaction step.

Compound S1.8-A3 tert-butyl (2-(2-(2-(4-amino-2-butyl-1H-imidazo[4,5-c]quinolin-1-yl)ethoxy)ethoxy)ethyl)carbamate, ^{1}H NMR (300 MHz, $CDCl_3$) δ 7.90 (d, *J* = 8.1 Hz, 1H), 7.81 (d, *J* = 8.3 Hz, 1H), 7.48 (t, *J* = 7.7 Hz, 1H), 7.29 (t, *J* = 6.3 Hz, 1H), 5.51 (s, 2H), 5.02 (s, 1H), 4.66 (t, *J* = 5.6 Hz, 2H), 3.94 (t, *J* = 5.6 Hz, 2H), 3.45 (s, 4H), 3.38 (t, *J* = 5.0 Hz, 2H), 2.99 – 2.91 (m, 2H), 1.97 – 1.76 (m, 2H), 1.58 – 1.45 (m, 2H), 1.43 (s, 9H), 0.99 (t, *J* = 7.3 Hz, 3H), ^{13}C NMR (75 MHz, $CDCl_3$) δ 154.64, 151.33, 144.87, 133.27, 127.44, 127.02, 126.95, 122.26, 119.50, 115.61, 77.58, 77.36, 77.16, 76.74, 71.16, 70.45, 70.28, 69.68, 64.42, 45.70, 30.11, 28.55, 27.32, 25.51, 22.78, 14.01, white solid, yield 2.3 g (32% over two steps of synthesis), MS $(M+H)^+$ calcd *m/z* 472.33, found *m/z* 472.29. Compound S1.7-A3 dissolved in 5 mL of acetic acid and subsequently a spatula tip of Zn powder was added. The next day, another spatula tip of Zn powder and 5 mL of acetic acid were added. The day after 7 mL of acetic acid, 40 mL of water and 1 spatula of Zn powder. On the fourth day another spatula of Zn powder was added, followed by 4 mL acetic acid and 2 spatula of Zn powder on fifth day. Reaction was completed on fifth day, diluted with 100 mL water and underlayered with 200 mL dichloromethane. Mixture was vigorously stirred, cooled with ice and potassium hydroxide added until Zn formed a soluble complex. Product was extracted with liquid-liquid extraction (three times with dichloromethane). Organic phases were combined, washed with water and brine, dried with sodium sulphate and solvent was evaporated under vacuum to obtain 7 g of crude product. To purify substance flash chromatography was performed with a gradient of cyclohexane and isopropanol (with 0.25% triethylamine each) to obtain 2.3 g of compound S1.8-A3.

Compound A3 1-(2-(2-(2-aminoethoxy)ethoxy)ethyl)-2-butyl-1H-imidazo[4,5-c]quinolin-4-amine, ^{1}H NMR (300 MHz, $CDCl_3$) δ 7.89 (d, *J* = 8.2 Hz, 1H), 7.79 (d, *J* = 7.7 Hz, 1H), 7.50 – 7.42 (m, 1H), 7.34 – 7.20 (m, 1H), 5.87 – 5.10 (m, 2H), 4.64 (t, *J* = 5.6 Hz, 2H), 3.94 (dd, *J* = 10.2, 4.6 Hz, 2H), 3.33 (t, *J* = 5.2 Hz, 2H), 2.94 (dd, *J* = 9.9, 5.9 Hz, 2H), 2.80 – 2.68 (m, 2H), 2.14 (s, 2H), 1.85 (dt, *J* = 15.5, 7.6 Hz, 3H), 1.48 (tq, *J* = 13.9, 6.9 Hz, 4H), 0.98 (t, *J* = 7.3 Hz, 4H), ^{13}C NMR (75 MHz, $CDCl_3$) δ 154.53, 151.28, 144.76, 127.33, 126.97, 122.22, 119.52, 115.56, 73.60, 71.12, 70.35, 69.68, 64.27, 45.68, 41.78, 30.08, 27.27, 25.48, 22.76, 13.98, white solid, yield 1 g (98%), purity 97% via HPLC-ELSD, MS $(M+H)^+$ calcd *m/z* 372.24, found *m/z* 372.67. Compound S1.8-A3 (1.29 g, 2.75 mmol) was dissolved in a mixture of dichloromethane and trifluoroacetic acid (10:1, 10 mL) and stirred at RT. After 30 min reaction was completed. Solution was poured into water and neutralized with potassium hydroxide. Product was extracted with liquid-liquid extraction (three times with dichloromethane). Organic phases were combined, washed with water and brine, dried with sodium sulphate and solvent was evaporated under vacuum to obtain 1 g of A3.

Compound S1.4-A4 2-(2-(2-((2-chloro-3-nitroquinolin-4-yl)amino)ethoxy)ethoxy)ethan-1-ol, white solid, yield 2.5 g (57%), MS $(M+H)^+$ calcd *m/z* 356.10, found *m/z* 356.33. Compound S1.3 (3 g, 12.4 mmol) was dissolved in 10 mL of anhydrous dichloromethane with triethylamine (2.57 mL, 1.5 eq). To another 5 mL of anhydrous dichloromethane 2.22 g of tert-butyl (2-(2-(2-aminoethoxy)ethoxy)ethyl)carbamate (SynInnova SI-551) was added. Both solutions were combined and heated to 45 °C for 3 days. Solvents were removed under vacuum. Crude product was then purified via column chromatography on silica gel (300 g) with a gradient of cyclohexane and acetone (with 0.2% of triethylamine each) to obtain 2.5 g of S1.4-A4.

Compound S1.5-A4 2-(2-(2-((3-amino-2-chloroquinolin-4-yl)amino)ethoxy)ethoxy)ethan-1-ol, white solid, yield 2.2 g (96%), MS $(M+H)^+$ calcd *m/z* 326.13, found *m/z* 326.40. Compound S1.4-A4 (2.5 g) was dissolved in ethyl acetate in a closed vessel. Vessel was flushed with Ar (evaporated and

flushed three times three times). To solution a spatula of sodium sulphate and 500 mg of Pt/C (5% Pt, Sigma-Aldrich 205931-1G). The vessel was again flushed three times with Ar. Subsequently the vessel was flushed with H_2 (Air Liquide 130415). After five days reaction was completed, solution filtered through celite and solvent removed under vacuum to yield 2.2 g of S1.5-A4 and used without further purification.

Compound S1.6-A4 2-(2-(2-(2-butyl-4-chloro-1H-imidazo[4,5-c]quinolin-1-yl)ethoxy)ethoxy)ethyl pentanoate, brown oil, yield 2.1 g (65%), MS $(M+H)^+$ calcd *m/z* 476.23, found *m/z* 476.47. Compound S1.5-A4 (2.2 g, 6.77 mmol) was dissolved in 5 mL trimethyl orthovalerate (VWR L20377.22) and solution was heated to 135 °C. After two hours, the starting material was completely depleted. The product and a methoxy by-product (m/z = 508) were formed. Methoxy group and trimethyl orthovalerate residues were removed under vacuum (approx. 80 mbar) at 140 °C to yield 2.1 g of S1.6-A4.

Compound S1.7-A4 2-(2-(2-(2-butyl-4-hydrazineyl-1H-imidazo[4,5-c]quinolin-1-yl)ethoxy)ethoxy)ethan-1-ol, MS $(M+H)^+$ calcd *m/z* 388.23, found *m/z* 388.47. Compound S1.6-A4 (2.1 g, 4.42 mmol) was dissolved in 10 mL of ethanol, subsequently hydrazine (Sigma-Aldrich 225819-500ML, 2 mL, 63 mmol) was added. Solution was refluxed overnight. MS analysis showed full conversion. Product S1.7-A4 was directly used for next reaction step.

Compound A4 2-(2-(2-(4-amino-2-butyl-1H-imidazo[4,5-c]quinolin-1-yl)ethoxy)ethoxy)ethan-1-ol, ^{1}H NMR (300 MHz, $CDCl_3$) δ 7.83 (dd, *J* = 18.0, 8.3 Hz, 2H), 7.46 (t, *J* = 7.7 Hz, 1H), 7.26 (dd, *J* = 8.8, 6.4 Hz, 1H), 5.68 (s, 2H), 4.62 (t, *J* = 5.5 Hz, 2H), 3.91 (t, *J* = 5.5 Hz, 2H), 3.69 – 3.61 (m, 2H), 3.50 – 3.39 (m, 6H), 2.97 – 2.88 (m, 2H), 1.83 (dt, *J* = 15.6, 7.6 Hz, 2H), 1.47 (dq, *J* = 14.6, 7.3 Hz, 2H), 0.97 (t, *J* = 7.3 Hz, 3H), ^{13}C NMR (75 MHz, $CDCl_3$) δ 154.92, 151.31, 144.63, 133.24, 127.19, 127.03, 126.79, 122.25, 119.49, 115.46, 72.87, 71.25, 70.40, 69.59, 61.58, 45.70, 30.01, 27.29, 22.74, 13.98, white solid, yield 967 mg (59% over two steps of synthesis), purity >99% via HPLC-ELSD, MS $(M+H)^+$ calcd *m/z* 373.22, found *m/z* 373.53. Compound S1.7-A4 dissolved in 10 mL of acetic acid and 10 mL of water and cooled with ice. Subsequently two spatula tips of Zn powder were added. After 30 min another spatula tip of Zn powder was added. After 4 h 10 mL of acetic acid, 10 mL of water and one spatula tip of Zn were added. The next day, reaction was completed. Solution was diluted with 100 mL water and underlayered with 200 mL dichloromethane. Mixture was vigorously stirred, cooled with ice and potassium hydroxide added. Product was extracted with liquid-liquid extraction (three times with dichloromethane). Organic phases were combined, washed with water and brine, dried with sodium sulphate and solvent was evaporated under vacuum to obtain 1.67 g of crude product. To purify substance flash chromatography was performed (Interchim puriFlash 5.020 with Interchim PF-15SIHP-F0040 column) with a gradient of cyclohexane, dichloromethane and ethanol to obtain 967 mg of compound A4.

Compound P1 (2R,3S,4R,5R,8R,10R,11R,12S,13S,14R)-2-ethyl-3,4,10-trihydroxy-13-(((2R,4R,5S,6S)-5-hydroxy-4-methoxy-4,6-dimethyltetrahydro-2H-pyran-2-yl)oxy)-11-(((2S,3R,4S,6R)-3-hydroxy-6-methyl-4-(methylamino)tetrahydro-2H-pyran-2-yl)oxy)-3,5,6,8,10,12,14-heptamethyl-1-oxa-6-azacyclopentadecan-15-one, ^{1}H NMR (400 MHz, MeOD) δ 5.07 (d, *J* = 4.8 Hz, 1H), 4.92 (dd, *J* = 10.3, 2.4 Hz, 1H), 4.55 (d, *J* = 7.6 Hz, 1H), 4.26 (ddd, *J* = 15.6, 8.2, 3.9 Hz, 2H), 3.88 – 3.78 (m, 1H), 3.70 (d, *J* = 7.1 Hz, 1H), 3.59 (d, *J* = 4.1 Hz, 1H), 3.40 (s, 3H), 3.34 (dt, *J* = 3.3, 1.6 Hz, 1H), 3.14 – 3.04 (m, 2H), 2.89 – 2.77 (m, 2H), 2.60 (ddd, *J* = 10.1, 9.4, 3.4 Hz, 2H), 2.45 (d, *J* = 10.5 Hz, 1H), 2.41 (s, 3H), 2.34 (s, 3H), 2.19 (t, *J* = 11.7 Hz, 1H), 2.09 – 1.96 (m, 3H), 1.94 – 1.84 (m, 1H), 1.80 (d, *J* = 14.7 Hz, 1H), 1.63 (dd, *J* = 15.1, 5.0 Hz, 1H), 1.57 – 1.46 (m, 1H), 1.45 – 1.37 (m, 1H), 1.36 (s, 2H), 1.33 (d, *J* = 6.2 Hz, 3H), 1.29 (s, 3H), 1.25 (d, *J* =

7.5 Hz, 3H), 1.21 (d, *J* = 6.0 Hz, 3H), 1.15 – 1.06 (m, 9H), 0.93 (dd, *J* = 13.0, 7.1 Hz, 5H), ^{13}C NMR (101 MHz, MeOD) δ 180.08, 103.24, 96.28, 84.24, 79.48, 79.45, 78.15, 76.00, 75.57, 75.48, 75.12, 74.36, 70.88, 68.76, 66.49, 63.61, 61.28, 50.04, 46.82, 43.61, 43.39, 38.03, 36.80, 35.91, 33.18, 28.14, 27.76, 22.33, 22.18, 21.92, 21.68, 19.04, 17.36, 15.46, 11.49, 9.78, 7.65, colorless solid crystals, yield 10 g (51%), MS $(M+H)^+$ calcd *m/z* 735.50, found *m/z* 735.80. P1 was described earlier in WO 03/070173 A2 (Burnet et al.). Briefly, Azithromycin (Atomaxchemicals AM83905015, 20 g, 26.7 mmol) and sodium hydrogen carbonate (6 g, 71.5 mmol) were dissolved in 120 mL of Methanol in a 500 mL Erlenmeyer flask. Potassium carbonate (12 g, 87 mmol) was dissolved in 80 mL of water. The potassium carbonate solution and the iodine (6.3 g, 24.8 mmol) were added, and the mixture was stirred vigorously at RT, until the dark color has disappeared. A second batch of iodine (6.3 g, 24.8 mmol) and solid potassium carbonate (4.2 g, 30.4 mmol) were added. This procedure was repeated once, until MS shows full conversion ($[M+H]^+$ = 735). Sodium bisulfite (Fisher 10474511, 1 g, 10 mmol) was added to remove excess oxidants, and all volatiles were evaporated. The solid residue is finely ground and extensively extracted by Soxhlet extraction with acetonitrile. The extract is concentrated to approx. 75ml and left standing at RT at least for 1 day, following another day in the fridge. All solids were collected and recrystallized from methanol with addition of ca. 1-2 mL of water. Crystallization proceeded for about 3 days in an open vessel to yield 10 g (51%) of large crystals and used without further purification.

Compound P2 N-(2-(((2S,3R,4S,6R)-2-(((2R,3S,4R,5R,8R,10R,11R,12S,13S,14R)-2-ethyl-3,4,10-trihydroxy-13-(((2R,4R,5S,6S)-5-hydroxy-4-methoxy-4,6-dimethyltetrahydro-2H-pyran-2-yl)oxy)-3,5,6,8,10,12,14-heptamethyl-15-oxo-1-oxa-6-azacyclopentadecan-11-yl)oxy)-3-hydroxy-6-methyltetrahydro-2H-pyran-4-yl)(methyl)amino)-2-oxoethyl)-N-methylglycine, ^{1}H NMR (400 MHz, DMSO) δ 4.85 (s, 1H), 4.76 (d, *J* = 8.1 Hz, 1H), 4.45 (d, *J* = 6.9 Hz, 1H), 4.38 (s, 1H), 4.14 (s, 1H), 4.11 – 4.00 (m, 1H), 3.75 (d, *J* = 14.4 Hz, 1H), 3.63 (d, *J* = 14.7 Hz, 1H), 3.49 (dd, *J* = 16.0, 10.9 Hz, 3H), 3.38 (dd, *J* = 14.0, 7.0 Hz, 1H), 3.31 – 3.21 (m, 5H), 3.22 – 3.07 (m, 1H), 2.98 – 2.88 (m, 1H), 2.85 (s, 1H), 2.75 (dd, *J* = 20.7, 13.9 Hz, 1H), 2.70 (s, 2H), 2.61 (s, 1H), 2.50 (s, 1H), 2.43 (d, *J* = 13.6 Hz, 3H), 2.26 (s, 4H), 1.90 (s, 2H), 1.78 (dd, *J* = 11.6, 7.4 Hz, 1H), 1.67 (d, *J* = 8.3 Hz, 1H), 1.51 (dd, *J* = 22.3, 11.9 Hz, 2H), 1.36 (d, *J* = 8.0 Hz, 3H), 1.16 (dd, *J* = 18.5, 10.9 Hz, 9H), 1.09 (dd, *J* = 6.1, 3.9 Hz, 7H), 1.01 (d, *J* = 7.2 Hz, 3H), 1.00 – 0.91 (m, 5H), 0.86 (d, *J* = 6.1 Hz, 3H), 0.79 (t, *J* = 7.3 Hz, 3H). ^{13}C NMR (101 MHz, DMSO) δ 177.03, 102.14, 94.46, 77.35, 76.27, 73.63, 72.78, 72.69, 69.65, 66.57, 64.83, 64.74, 58.22, 58.07, 48.92, 48.75, 44.78, 44.63, 42.00, 41.88, 41.66, 41.58, 40.14, 39.94, 39.73, 39.52, 39.31, 39.10, 38.90, 35.76, 34.56, 27.07, 26.40, 25.93, 22.01, 21.07, 20.98, 20.94, 18.43, 18.33, 17.63, 15.07, 14.74, 14.65, 10.88, 9.03, 6.83, colorless solid, yield 1.89 g (80%), MS $(M+H)^+$ calcd *m/z* 864.54, found *m/z* 864.73. Compound P1 (2.00 g, 2.72 mmol) and 4-Methylmorpholine-2,6-dione (Sigma-Aldrich 734217-1G, 369 mg, 2.86 mmol) were solved in DMF (25 mL). Reaction showed full conversion via MS and TLC (acetone with 7% triethylamine) after 15 min. Polystyrene-NH_2 (Iris Biotech SR-1132, 78 mg) was added to remove the excess of 4-Methylmorpholine-2,6-dione and stirred for 30 min. Polystyrene was then filtered off, solvents removed under vacuum to yield 1.89 g of P2 and used without further purification.

Compound A1-Mac 2-((2-(4-(2-(4-amino-2-butyl-1H-imidazo[4,5-c]quinolin-1-yl)ethyl)piperidin-1-yl)-2-oxoethyl)(methyl)amino)-N-((2S,3R,4S,6R)-2-(((2R,3S,4R,5R,8R,10R,11R,12S,13S,14R)-2-ethyl-3,4,10-trihydroxy-13-(((2R,4R,5S,6S)-5-hydroxy-4-methoxy-4,6-dimethyltetrahydro-2H-pyran-2-yl)oxy)-3,5,6,8,10,12,14-heptamethyl-15-oxo-1-oxa-6-azacyclopentadecan-11-yl)oxy)-3-hydroxy-6-methyltetrahydro-2H-pyran-4-yl)-N-methylacetamide, ^{1}H NMR (400 MHz, $CDCl_3$) δ 7.82 (t, *J* = 7.6 Hz, 2H), 7.48 (t, *J* = 7.7 Hz, 1H), 7.30 (t, *J* = 7.5 Hz, 1H), 5.84 (s, 1H), 5.29 – 5.00 (m, 2H), 4.74 – 4.55 (m, 3H), 4.48 (t, *J* = 7.7 Hz, 1H), 4.43 – 4.32 (m, 2H), 4.19 (t, *J* = 6.4 Hz, 1H), 4.14 – 3.97 (m, 2H), 3.64 (dd, *J* = 18.2, 12.6 Hz, 4H), 3.55 (dd, *J* = 14.6, 7.2 Hz, 1H), 3.44 – 3.36 (m,

3H), 3.36 – 3.32 (m, 4H), 3.32 – 3.23 (m, 3H), 3.11 – 2.99 (m, 3H), 2.94 (d, *J* = 3.6 Hz, 2H), 2.86 – 2.80 (m, 3H), 2.67 (d, *J* = 7.1 Hz, 2H), 2.53 (d, *J* = 9.9 Hz, 1H), 2.42 (t, *J* = 7.9 Hz, 3H), 2.30 (s, 4H), 2.04 – 1.94 (m, 3H), 1.83 (dt, *J* = 17.8, 8.2 Hz, 7H), 1.69 (d, *J* = 14.4 Hz, 2H), 1.52 – 1.37 (m, 5H), 1.33 (s, 2H), 1.31 (d, *J* = 4.2 Hz, 5H), 1.24 – 1.19 (m, 7H), 1.16 (ddd, *J* = 11.9, 6.9, 3.6 Hz, 6H), 1.07 (d, *J* = 6.1 Hz, 6H), 1.02 – 0.94 (m, 7H), 0.89 (d, *J* = 6.6 Hz, 3H), 0.85 (d, *J* = 7.5 Hz, 3H), ^{13}C NMR (101 MHz, $CDCl_3$) δ 178.65, 171.03, 168.62, 153.41, 151.13, 133.38, 127.30, 126.74, 122.60, 119.34, 115.19, 78.23, 77.71, 77.48, 77.16, 76.84, 74.42, 73.81, 73.69, 73.25, 71.54, 71.27, 70.17, 68.47, 65.93, 65.84, 62.55, 54.42, 53.21, 49.62, 49.55, 45.32, 43.60, 43.36, 42.55, 41.99, 36.97, 36.81, 36.44, 35.82, 34.99, 34.37, 31.90, 30.16, 29.25, 27.47, 27.21, 27.10, 26.86, 22.71, 22.07, 21.74, 21.35, 21.14, 21.08, 18.47, 18.33, 16.37, 15.03, 13.96, 11.29, 9.58, 8.19, 7.53, white solid, yield 162 mg (35%), purity 96.1% via HPLC-ELSD, MS $(M+H)^+$ calcd *m/z* 1197.77, found *m/z* 1197.67, HRMS $(M+H)^+$ calcd *m/z* 1197.77448, found *m/z* 1197.77511, $(M+Na)^+$ calcd *m/z* 1219.75642, found *m/z* 1219.75708. Starting from A1 (135 mg, 0.385 mmol) the coupling with P2 (350 mg, 1.05 eq) using (1-[Bis(dimethylamino)methylene]-1H-1,2,3-triazolo[4,5-b]pyridinium 3-oxide hexafluorophosphate (HATU, Fluorochem 50215, 161 mg, 1.1 eq) and triethylamine (80 µL, 1.5 eq) was carried out in dichloromethane. Reaction was complete after 3 h (reaction progress checked via MS). Solvents were evaporated and crude product was purified via flash chromatography with a gradient of cyclohexane, isopropanol and dichloromethane to yield 162 mg of A1-Mac.

Compound A2-Mac 2-((2-(4-(2-(4-amino-2-butyl-1H-imidazo[4,5-c]quinolin-1-yl)ethyl)piperidin-1-yl)-2-oxoethyl)(methyl)amino)-N-((2S,3R,4S,6R)-2-(((2R,3S,4R,5R,8R,10R,11R,12S,13S,14R)-2-ethyl-3,4,10-trihydroxy-13-(((2R,4R,5S,6S)-5-hydroxy-4-methoxy-4,6-dimethyltetrahydro-2H-pyran-2-yl)oxy)-3,5,6,8,10,12,14-heptamethyl-15-oxo-1-oxa-6-azacyclopentadecan-11-yl)oxy)-3-hydroxy-6-methyltetrahydro-2H-pyran-4-yl)-N-methylacetamide, ^{1}H NMR (400 MHz, $CDCl_3$) δ 7.82 (t, *J* = 6.2 Hz, 2H), 7.50 (t, *J* = 7.6 Hz, 1H), 7.31 (t, *J* = 7.6 Hz, 1H), 5.86 (s, 1H), 5.10 (s, 2H), 4.73 – 4.66 (m, 1H), 4.63 (d, *J* = 10.8 Hz, 2H), 4.51 – 4.42 (m, 1H), 4.29 (s, 2H), 4.20 (s, 1H), 4.06 (s, 2H), 3.66 (d, *J* = 18.9 Hz, 4H), 3.56 (dd, *J* = 14.6, 7.3 Hz, 2H), 3.37 – 3.30 (m, 7H), 3.03 (d, *J* = 6.9 Hz, 2H), 2.92 (s, 2H), 2.88 – 2.81 (m, 4H), 2.75 – 2.62 (m, 3H), 2.53 (d, *J* = 9.7 Hz, 2H), 2.44 – 2.37 (m, 4H), 2.31 (s, 4H), 1.85 (dt, *J* = 15.6, 7.7 Hz, 3H), 1.74 – 1.66 (m, 2H), 1.46 (dt, *J* = 20.3, 7.3 Hz, 6H), 1.32 (d, *J* = 7.8 Hz, 9H), 1.25 – 1.19 (m, 10H), 1.07 (s, 5H), 0.98 (t, *J* = 7.5 Hz, 7H), 0.90 (d, *J* = 5.6 Hz, 4H), 0.86 (t, *J* = 7.4 Hz, 6H), ^{13}C NMR (101 MHz, $CDCl_3$) δ 178.64, 171.01, 170.49, 168.66, 154.06, 151.34, 133.39, 127.23, 127.06, 122.49, 119.46, 115.45, 78.21, 77.73, 77.48, 77.16, 76.84, 74.42, 73.80, 73.69, 73.27, 71.57, 70.23, 68.52, 65.96, 65.82, 62.54, 59.35, 54.42, 53.19, 50.80, 49.54, 45.31, 43.34, 42.56, 41.69, 37.40, 36.46, 35.81, 34.97, 30.15, 29.79, 29.75, 29.45, 29.24, 27.61, 27.46, 27.09, 26.87, 22.70, 22.08, 21.73, 21.33, 21.09, 18.47, 18.33, 16.36, 15.06, 13.98, 11.30, 9.59, 8.20, 7.52, white solid, yield 143 mg (32%), purity >99% via HPLC-ELSD, MS $(M+H)^+$ calcd *m/z* 1183.76, found *m/z* 1184.07, HRMS $(M+H)^+$ calcd *m/z* 1183.75883, found *m/z* 1183.75812, $(M+Na)^+$ calcd *m/z* 1205.74077, found *m/z* 1205.74005. Starting from A2 (127 mg, 0.377 mmol) the coupling with P2 (376 mg, 1.1 eq) using HATU (Fluorochem 50215, 158 mg, 1.1 eq) and triethylamine (78 µL, 1.5 eq) was carried out in dichloromethane. Reaction was complete after 5 h (reaction progress checked via MS). Solvents were evaporated and crude product was purified via flash with a gradient of cyclohexane, isopropanol and dichloromethane to yield 143 mg of A2-Mac.

2. HPLC-MS/MS

Table S1. Specific detection parameters of substances in HPLC-MS/MS. A1-mac and A2-mac were analyzed as their double charged ion $(M+2H)^{2+}$. Terbuthylazine was used as internal standard for calibration of HPLC-MS/MS.

Substance	Q1 mass	Q3 mass	DP (V)	CE (V)	CXP (V)
RSQ	315	197	106	43	36
IMQ	241	185	96	33	36
A1	352	241	56	35	14
A1-mac	600	521	91	31	34
A2-mac	592	421	116	49	26
Terbuthylazine	230	174	61	23	10

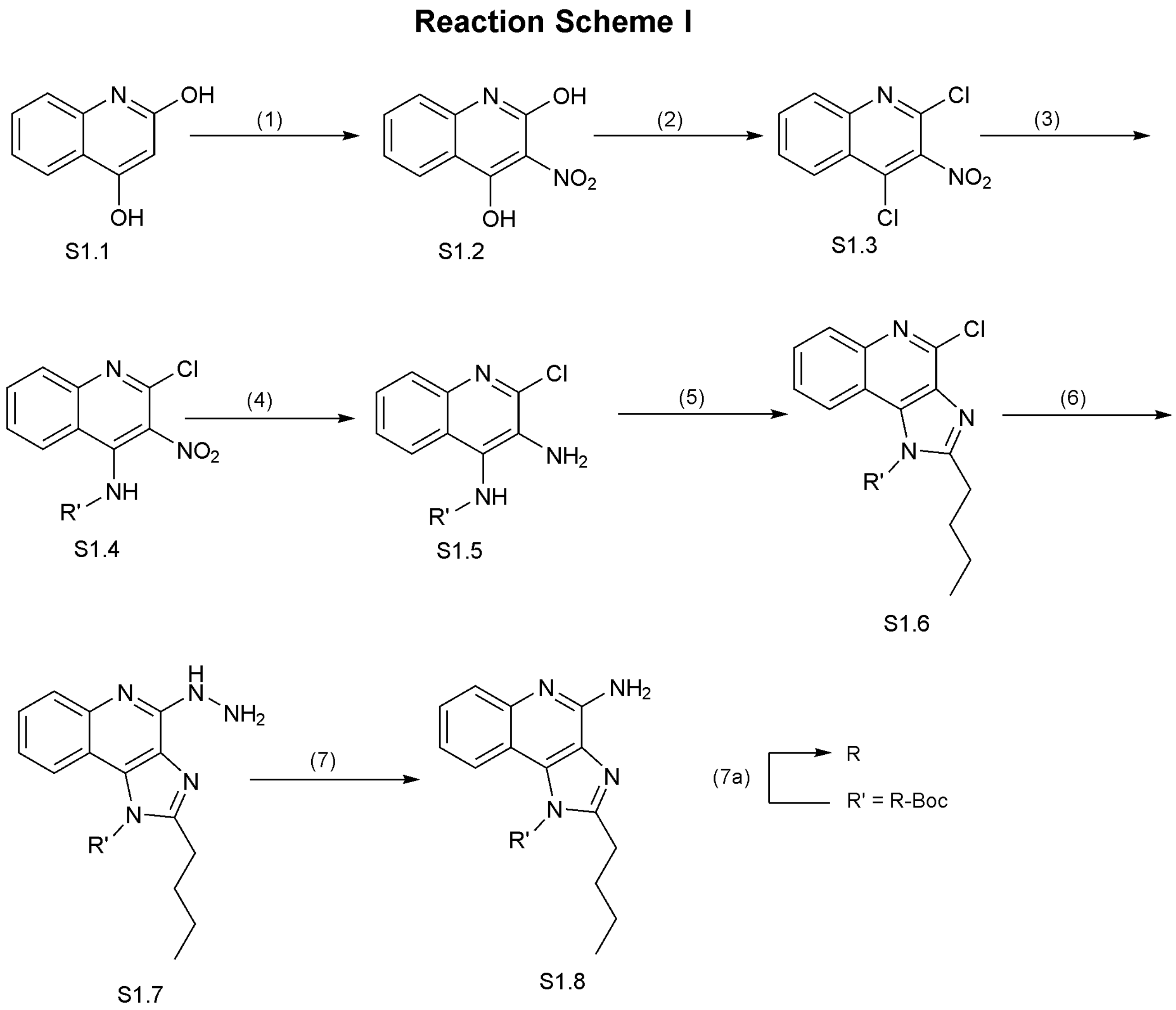

FIGURE S1 | Reagents and conditions: (1) HNO_3, (2) $PhP(=O)Cl_2$, (3) H_2N-R’, 45°C, (4) H_2/Pd or $Na_2S_2O_4$, (5) $C_4H_9C(OCH_3)_3$, Δ, (6) N_2H_4/H_2O, Δ, (7) Zn/TFA/EtOH, (7a) DCM/TFA; deprotection of Boc group, if present. R can be 2-(4-piperidinyl)ethyl (for compound A1) and (4-piperidinyl)methyl (for compound A2), 2-[2-(2-aminoethoxy)ethoxy]ethyl (for compound A3). For compound A4 R’ is 2-[2-(2-hydroxyethoxy)ethoxy]ethyl (in steps S14 to S.7 as pentanoate).

Reaction Scheme II

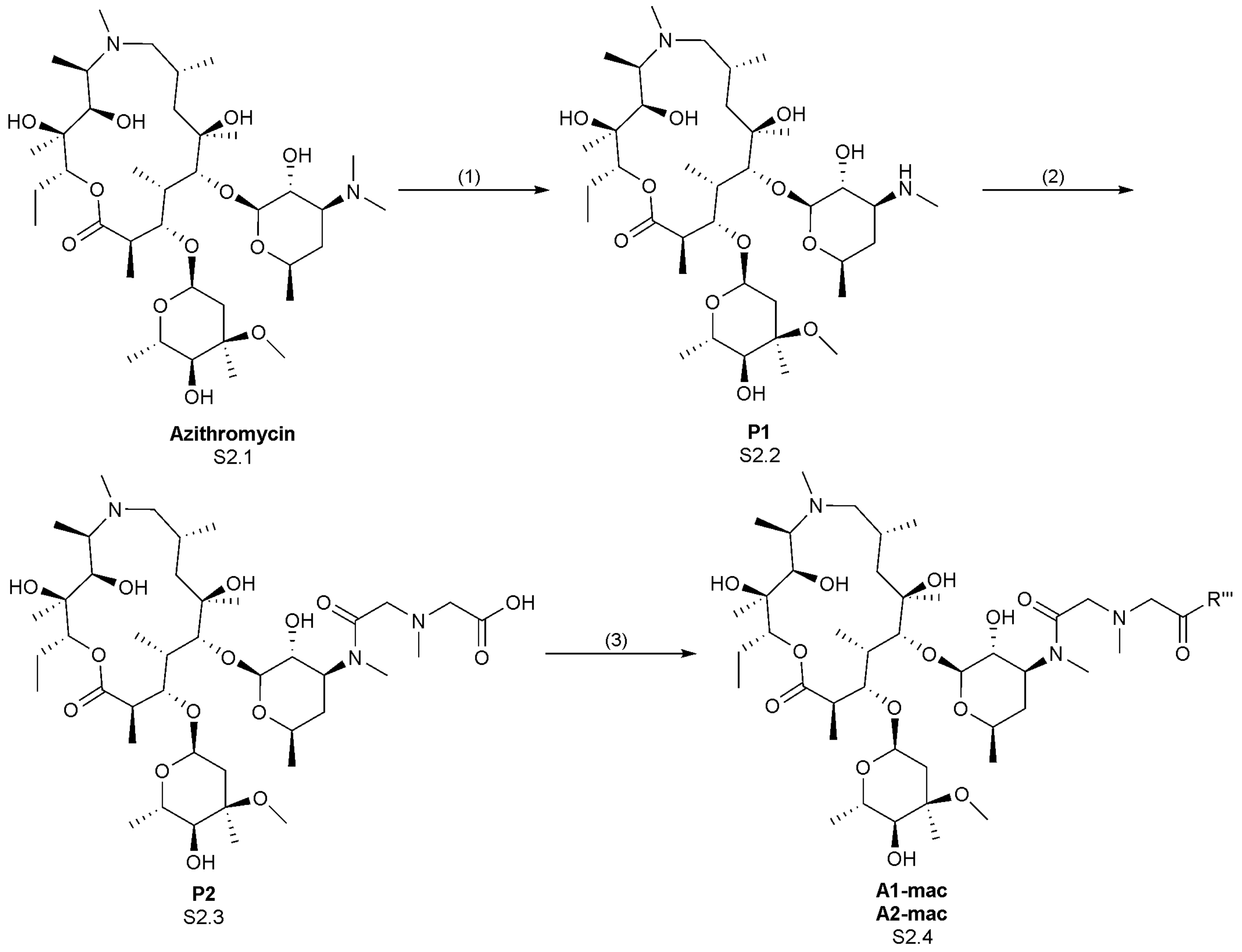

FIGURE S2 | Reagents and conditions: (1) Iodine, K_2CO_3, (2) 4-Methylmorpholine-2,6-dione, (3) HATU, triethylamine; R''' can be A1 or A2.

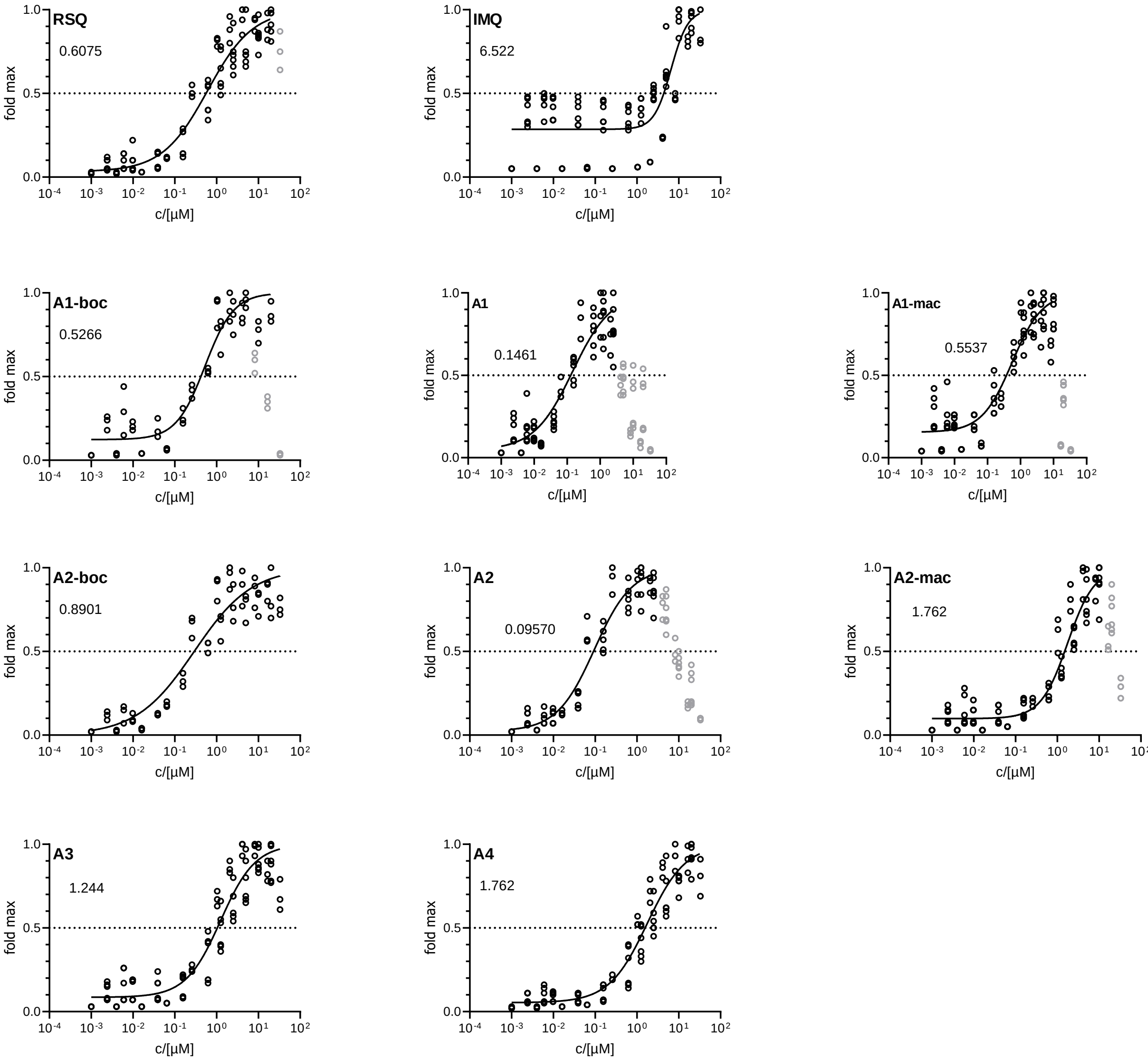

FIGURE S3 | Dose response of compounds in HEK Blue hTLR7. Circles represent normalized replicate measurements pooled from 3 experiments. Lines represent non-linear functions fit to the data to calculate compound-specific EC50, values in grey have been excluded before fitting.

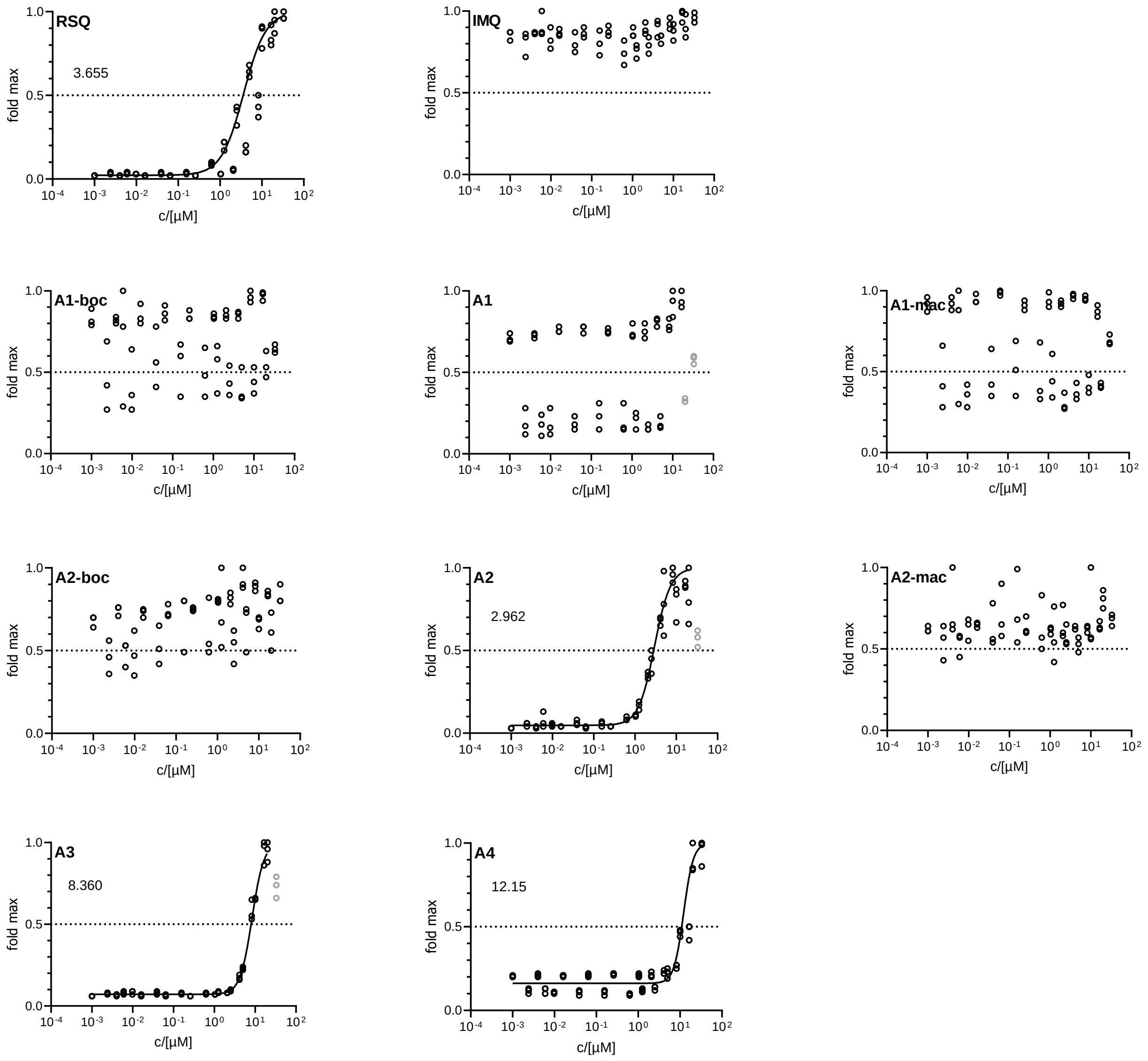

FIGURE S4 | Dose response of compounds in HEK Blue hTLR8. Circles represent normalized replicate measurements pooled from 3 experiments. Lines represent non-linear functions fit to the data to calculate compound-specific EC50, values in grey have been excluded before fitting.

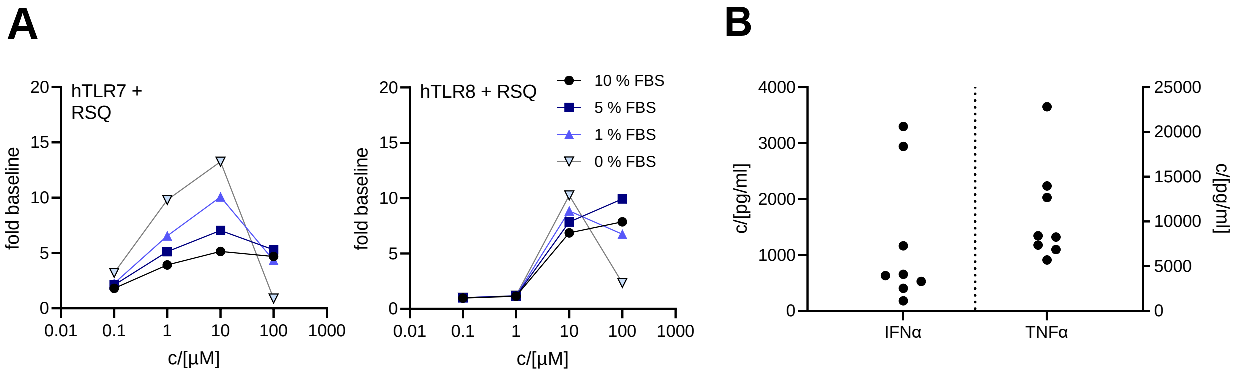

FIGURE S5 | A Relationship between serum concentration, signal to noise ratio and compound toxicity at high doses in the HEK Blue hTLR7/8 reporter assay **B** Variance in maximal cytokine secretion between donors in the full blood stimulation assay. Each point represents the maximal concentration of a given cytokine observed for a donor (for TNFα (right axis) generally samples treated with 10 µM RSQ, for IFNα (left axis) samples treated with either 10 µM RSQ or A1-mac), n=8

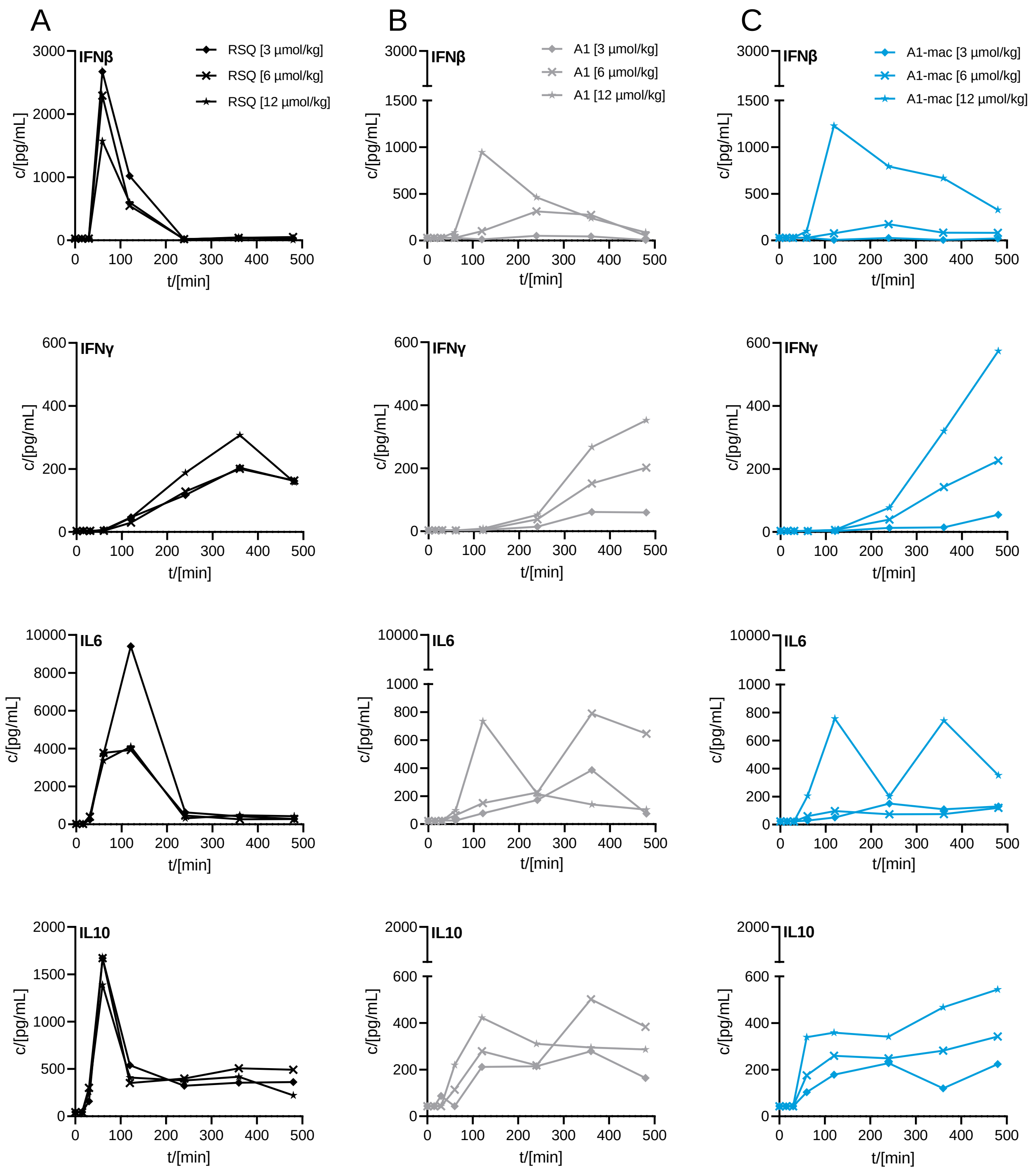

FIGURE S6 | Cytokine profile in peripheral blood over time after 3, 6 and 12 µmol/kg s.c. compound administration in 8-week-old, female C57BL/6 mice (n=3 mice per group). IFNβ, IFNγ, IL6, IL10 levels in tail plasma over time were determined via cytometric bead array. At each sampling timepoint the plasma of mice in one treatment group **(A)** RSQ **(B)** A1 **(C)** A1-mac was pooled.

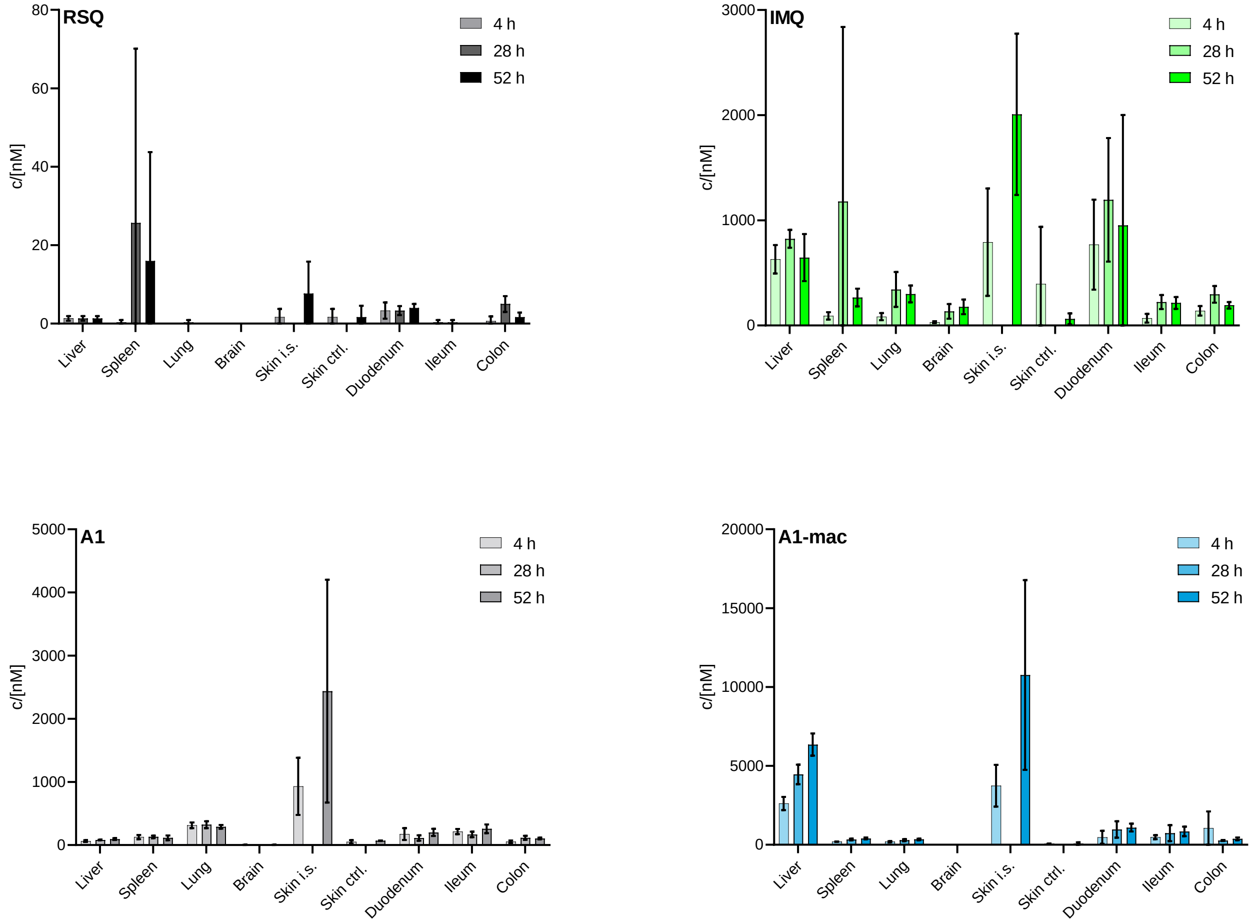

FIGURE S7 | Compound concentration after repeated applications of A1, A1-mac compared to RSQ and IMQ. Samples were collected 4 h, 28 h and 52 h after s.c. treatment. Skin samples were sampled at time points 4 h and 52 h. Compound concentrations were analyzed by HPLC-MS/MS, data presented as mean ± SD
